# Jumu is required for circulating hemocyte differentiation and phagocytosis in *Drosophila*

**DOI:** 10.1186/s12964-018-0305-3

**Published:** 2018-12-05

**Authors:** Yangguang Hao, Shichao Yu, Fangzhou Luo, Li Hua Jin

**Affiliations:** 1 Department of Genetics, College of Life Sciences, Northeast Forestry University, Harbin, 150040 People’s Republic of China; 2 Department of Translational medicine research center, Shenyang Medical College, Shenyang, 110034 People’s Republic of China

**Keywords:** *Drosophila*, Hemocytes, Phagocytosis, Cytoskeleton reorganization, Jumu

## Abstract

**Background:**

The regulatory mechanisms of hematopoiesis and cellular immunity show a high degree of similarity between insects and mammals, and *Drosophila* has become a good model for investigating cellular immune responses. Jumeau (Jumu) is a member of the winged-helix/forkhead (FKH) transcription factor family and is required for *Drosophila* development. Adult *jumu* mutant flies show defective hemocyte phagocytosis and a weaker defense capability against pathogen infection. Here, we further investigated the role of *jumu* in the regulation of larval hemocyte development and phagocytosis.

**Methods:**

In vivo phagocytosis assays, immunohistochemistry, Real-time quantitative PCR and immunoblotting were performed to investigate the effect of Jumu on hemocyte phagocytosis. 5-Bromo-2-deoxyUridine (BrdU) labeling, phospho-histone H3 (PH3) and TdT-mediated dUTP Nick-End Labeling (TUNEL) staining were performed to analyze the proliferation and apoptosis of hemocyte; immunohistochemistry and Mosaic analysis with a repressible cell marker (MARCM) clone analysis were performed to investigate the role of Jumu in the activation of Toll pathway.

**Results:**

Jumu indirectly controls hemocyte phagocytosis by regulating the expression of NimC1 and cytoskeleton reorganization. The loss of *jumu* also causes abnormal proliferation and differentiation in circulating hemocytes. Our results suggest that a severe deficiency of *jumu* leads to the generation of enlarged multinucleate hemocytes by affecting the normal cell mitosis process and induces numerous lamellocytes by activating the Toll pathway.

**Conclusions:**

Jumu regulates circulating hemocyte differentiation and phagocytosis in *Drosophila.* Our findings provide new insight into the mechanistic roles of cytoskeleton regulatory proteins in phagocytosis and establish a basis for further analyses of the regulatory mechanism of the mammalian ortholog of Jumu in mammalian innate immunity.

**Electronic supplementary material:**

The online version of this article (10.1186/s12964-018-0305-3) contains supplementary material, which is available to authorized users.

## Background

*Drosophila* lacks adaptive immunity and relies on multiple innate immune responses, such as humoral and cellular immunity, to defend against invading pathogens [1]. The humoral response mainly depends on systematic secretion of antimicrobial peptides (AMPs) by the fat body, and AMP synthesis is triggered and regulated by the Toll and Imd pathways [2, 3]. The cellular response is provided by the hemocyte lineage. The *Drosophila* hemocyte population consists of three broad subtypes of cells: plasmatocytes, crystal cells and lamellocytes [4, 5]. Plasmatocytes represent 90–95% of all mature larval circulating hemocytes and are involved in the phagocytosis of microbial pathogens, encapsulation of parasites and production of AMPs [1, 6, 7]. Crystal cells constitute 5% of larval hemocytes and participate in the melanization process during the encapsulation of invading organisms, wound repair and coagulation [8, 9]. Lamellocytes are rarely observed in healthy larvae but appear after parasitization and are involved in the encapsulation of foreign pathogens that are too large to undergo phagocytosis [7, 9].

In recent years, the mechanisms underlying the humoral innate immune response have been intensively investigated particularly in insects and mammals. However, many of the mechanisms and roles of the cellular immune response have yet to be determined. Phagocytosis is an important defense mechanism in cellular immunity involved in both innate and adaptive immunity and has been conserved throughout evolution. The first step in phagocytosis is microbial recognition. In *Drosophila*, several proteins have been identified as phagocytic recognition receptors, such as Scavenger receptor class C, type I (Sr-CI) [10], Down syndrome cell adhesion molecule (Dscam) [11], peptidoglycan recognition protein LC (PGRP-LC) [12] and the EGF-like repeat-containing proteins Nimrod C1 (NimC1) [13] and Eater [14]. These phagocytosis receptors can recognize various pathogens by binding to phagocytosis markers present on the surface of target pathogenic organisms. After binding target cells, the intracellular portion of phagocytosis receptors activates a signaling pathway that leads to rearrangement of the actin cytoskeleton. The plasma membrane of phagocytes then extends and surrounds their targets. Finally, target cells are incorporated into phagocytes as phagosomes and then ingested [15]. The dynamics of the actin network are required for phagocytosis, cell migration and adhesion, and many proteins have been suggested to be involved in the rearrangement of the actin cytoskeleton. Arp2/3 controls filament polymerization and depolymerization through interactions with regulatory proteins [16]. The Rho-family GTPases Rho, Rac and Cdc42 direct the formation of different cellular protrusions, such as filopodia, membrane ruffles or large lamellipodial extensions [17, 18]. Rho1 can promote hemocyte cell spreading and the formation of filopodia [19, 20]. *Drosophila* Profilin, which is encoded by the *chickadee* gene, is required for the formation of normal filopodial and lamellipodial extensions during wound repair [21]. Enabled (Ena)/VASP family proteins can protect the growing barbed ends from capping by binding to them, thereby allowing continuous filament elongation and positively regulating the number and length of filopodia [22–24]. Fascin (*Drosophila* Singed, Sn) is a conserved actin-binding protein that cross-links clustered actin filaments and converts them into stable, bundled filopodia [25–27]. However, although several phagocytic receptors and a number of components of the cytoskeletal regulatory networks have been identified, the signaling pathways involved in phagocytosis and the reorganization of the actin network have yet to be determined.

Jumeau (Jumu) is a member of the winged-helix/forkhead (Fkh) transcription factor family in *Drosophila* and contains a conserved winged-helix/forkhead domain (WH/FKH) for DNA binding. The gene is widely expressed in most organizations throughout development, such as brain lobes, imaginal discs, the CNS, salivary gland and the hindgut [28–30]. Jumu is required for neurogenesis as well as eye, wing, and bristle development [28, 29]. Homozygous null mutants of *jumu* die as embryos or young larvae, and fitness and fertility are impaired in heterozygote null mutants [29]. Jumu regulates nucleolar morphology and function as well as chromatin organization [30]. A recent study indicated that Jumu regulates cardiac progenitor specification by controlling the expression of receptors of the fibroblast growth factor and Wnt signaling pathways [31]. Additionally, our previous studies have shown that Jumu is also expressed in hemocytes and fat body, involved in proper bacterial phagocytosis and resistance in adult flies, and overexpression of *jumu* both in the fat body and hemocytes induces melanotic nodules by activating Toll signaling [32, 33]. Our recent study showed that Jumu plays crucial roles during *Drosophila* lymph gland hematopoiesis [34].

In this study, we show that Jumu is required for larval circulating hemocyte development as well as phagocytosis and filopodium formation. The loss of one *jumu* copy induces an increase in the number of hemocytes. The loss of two *jumu* copies can inhibit normal hemocyte mitosis by affecting spindle formation and cytokinesis, resulting in enlarged multinucleated hemocytes. The severe deficiency of *jumu* also induces the generation of lamellocytes through activation of the Toll signal pathway. Furthermore, we found that Jumu regulates hemocyte phagocytosis by affecting the expression of NimC1 and cytoskeletal reorganization and controls the formation of lamellipodia and filopodia by regulating the expression of Ena and Fascin.

## Methods

### *Drosophila* strains

The following fly stocks were used: *jumu*^*GE27806*^ was purchased from GenExel (Daejeon, South Korea). *Df(3R)Exel6157* and *UAS-jumu* were gifts from Alan M. Michelson [31]. *Hml-delta-Gal4 UAS-2xEGFP* was a gift from Utpal Banerjee [35]. *eaterGFP* was a gift from Mika Rämet [36]. *Dif*^*1*^ was a gift from Bruno Lemaitre. *UAS-jumu RNAi* (*jumu*^*GD4099*^), *UAS-ena RNAi*, *UAS-fascin RNAi*, *UAS-eater RNAi* and *UAS-NimC1 RNAi* were obtained from the Vienna *Drosophila* RNAi Center (VDRC). *Hml-Gal4* were obtained from the Tsinghua Fly Center. *UAS-Rho1*^*N19*^, *UAS-Rho1*^*v14*^, *UAS-Rac1 DN, UAS-Rac1 CA,* and *da-Gal4* were obtained from the Bloomington Stock Center. *ppl-Gal4* was obtained from Xun Huang [37]. The *w*^*1118*^ and *jumu* mutants were reared at 25 °C and, except for the hemocyte number comparison shown in Fig. 6b, the offspring of crosses involving RNAi lines or the *UAS-jumu* line and all controls of these crosses were reared at 29 °C.

### Transgenic constructs

To generate the *UAS-NimC1* strain, the full-length *NimC1-RA* coding sequence was PCR amplified and cloned into pUAST between the underlined restriction sites. Then, the transgenic flies were generated using standard methods.

### Circulating hemocyte counts

Larvae were staged according to procedures described previously [32]. Precisely staged late-wandering third-instar larvae were used to obtain hemocyte counts. Circulating hemocyte counts were performed as described previously [32, 38]. Briefly, 5–6 wandering larvae were opened via an incision at both the posterior and anterior ends in 20 μl Phosphate Buffered Saline (PBS), and their hemolymph was allowed to leak out. The hemocytes were then transferred to a Neubauer improved hemocytometer (Marienfeld) for counting of the cells. Counts were conducted in at least 50 larvae in each experiment. All counting assays were performed for at least three independent experiments.

### In vivo phagocytosis of hemocytes

In vivo phagocytosis assays of larvae were performed as described previously [32]. Briefly, the ventral side of third-instar larvae was injected with fluorescent latex beads (Thermo Fisher Scientific, F8821), Alexa Fluor 488-labeled heat killed spores of *B. bassiana*, dead fluorescein-conjugated *E. coli* (K-12), *S. aureus* and pHRodo*-E. coli* (1 mg/ml, 180–200 nl) (Thermo Fisher Scientific) using a Picospritzer III injector. After incubation for 1 h, the circulating hemocytes were collected from the injected larvae by ripping the larval cuticle near the posterior end in PBS solution containing 0.4% trypan blue (trypan blue is used to quench fluorescence of nonphagocytosed bacteria), and the hemocytes were then transferred and attached to a glass slide for 30 min. The cells were subsequently fixed at room temperature with 3.7% formaldehyde in PBS for 10 min and then washed three times for 5 min with PBS. The phagocytosis of latex beads and bacteria by the circulating blood cells was observed using a Zeiss Axioplan 2 microscope equipped with fluorescence optics. For each genotype, 500–1000 cells were counted using ImageJ software. All phagocytosis assays were performed in at least three independent experiments.

### Immunohistochemistry

For antibody staining, hemocytes were bled from third-instar larva and allowed to attach to a glass slide for 30 min. The cells were then fixed at room temperature with 3.7% formaldehyde in PBS for 10 min, pre-incubated in blocking solution (PBS with 0.1% Tween-20 and 5% goat serum) and incubated with primary antiserum diluted in blocking solution. The following primary antibodies were used: mouse anti-NimC1, mouse anti-L1 and mouse anti-H2 (gifts from I. Ando); rat anti-Jumu (made in our lab); mouse anti-α-tubulin (sigma); rabbit anti-PH3 (Millipore); mouse anti-Dorsal, mouse anti-Ena, mouse anti-Fascin, mouse anti-Rho1 and mouse anti-Profilin (Developmental Studies Hybridoma Bank); rabbit anti-Dif (gift from Dominique Ferrandon). Alexa Fluor 488-, Alexa Fluor 568- and Alexa Fluor 594-conjugated secondary antibodies (Thermo Fisher Scientific) were employed. For phalloidin staining, hemocytes were preincubated with PBST (PBS with 0.1% TritonX-100) for 5 min and then incubated with Alexa Fluor 488-labeled phalloidin (Thermo Fisher Scientific) diluted in PBS for 30 min. Images were obtained using a Zeiss Axioplan 2 microscope equipped with fluorescence optics. All staining was performed in at least three independent experiments.

### BrdU labeling and TUNEL staining

The BrdU labeling was performed according to previously described procedures [38]. Briefly, BrdU was diluted in standard fly food at 0.5 mg/ml (Sigma), and trypan blue was added to the fly food to identify whether the larvae eat the fly food. The third-instar larvae were maintained on this media for 4 h, and then, the dissected hemocytes from the third-instar larvae were fixed with 3.7% formaldehyde in PBS for 30 min and washed three times for 10 min each in PBS with 0.1% Tween-20. The samples were then treated with 3 M HCl for 30 min and washed three times in PBS with 0.1% Tween-20. After blocking the samples with blocking solution (PBS with 0.1% Tween-20 and 5% goat serum), the samples were stained with a mouse anti-BrdU antibody (Developmental Studies Hybridoma Bank), followed by mouse-TRITC secondary antibody staining and mounting. TUNEL staining was conducted using TUNEL solution according to the manufacturer’s instructions (Roche Biochemicals). At least three independent experiments were performed.

### MARCM clone analysis

The genotype of the strain used for the MARCM clone generation was as follows: hsFLP, UAS-GFP, actGal4; FRT42D, tubGal80. We crossed this strain with *UAS-jumu RNAi*. The embryo collections (1 h) were incubated for 10 h at 25 °C and subsequently shifted to 37 °C for 1 h to induce the MARCM clones.

### Transfection and immunoblotting

S2 cells (CVCL_Z232, Invitrogen, Cat#R690–07) were transfected with pMK33-Flag or the pMK33-Flag-*jumu* full CDS construct using the Effectene Transfection kit (Qiagen). Whole-cell extracts were prepared in lysis buffer containing 20 mM Tris (pH 7.6), 150 mM NaCl, 10% glycerol, 1% Triton X-100, 1 mM dithiothreitol (DTT), 2 mM EDTA, and protease inhibitors. Then, 30 μg of the lysate was loaded into a 12% SDS-PAGE gel, followed by electroblotting onto nitrocellulose membranes and probing with mouse anti-α-tubulin (1:500, Sigma), mouse anti-Ena, mouse anti-Fascin, mouse anti-Rho1 and mouse anti-Profilin (1:300, Developmental Studies Hybridoma Bank) for 2 h. The blot was subsequently probed with anti-mouse HRP-conjugated secondary antibodies for 1.5 h and detected using the ECL Plus detection system (Program). ImageJ was employed to measure the intensity values of the blots. Representative blots obtained from at least three independent experiments with similar results are presented.

### Image analysis and quantification

All images used for quantification were captured with a Zeiss Axioplan 2 microscope, and all analyses were performed using ImageJ. The mitotic index and BrdU index of the circulating hemocytes were determined by dividing the number of PH3-positive cells and BrdU-positive cells, respectively, by the total number of cells, and at least 500 cells were counted. The signal intensities of NimC1, Ena, Fascin, Roh1 and Profilin were defined as the average pixel intensity values in each cell (integrated intensity in one cell/area of the cell), and at least 200 cells were measured. For quantification of the fluorescence signal intensity, the fluorescent images were first converted to 8-bit images, and the total intensity value with an identical threshold was captured and measured with ImageJ. The freehand selection tool in ImageJ was used to capture and measure the area of the circulating hemocytes and lamellipodia. Filopodial length was quantified using the line tool of ImageJ with protrusions > 1 μm long being classified as filopodia. At least 50 cells were measured for the quantification of the filopodial length, and at least 100 cells were measured for the quantification of the lamellipodial area.

### Real-time quantitative PCR

The total RNA obtained from 6 to 8 dissected third-instar larvae or circulating hemocytes (from 600 to 800 third-instar larvae) was prepared using TRIzol (Invitrogen). The obtained total RNA was used to generate cDNA with M-MLV Reverse Transcriptase (Promega). Real-time PCR amplification was performed using SYBR Green I Master Mix (Roche, LightCycler480) on a Roche 480 real-time PCR system. The results were normalized to the level of *RpL32* mRNA in each sample. Three experiments per genotype were averaged. Two biological replicates were performed. The primer sequences used are shown in Additional file 1: Table S1.

### Statistical analysis

Statistical analyses were performed with two-tailed unpaired Student’s t-tests or one-way ANOVAs using GraphPad Prism software. The thresholds for statistical significance were established as *P < 0.05, **P < 0.01 and ***P < 0.001.

## Results

### Jumu is required for hemocyte phagocytosis and development

**Fig. 1:**
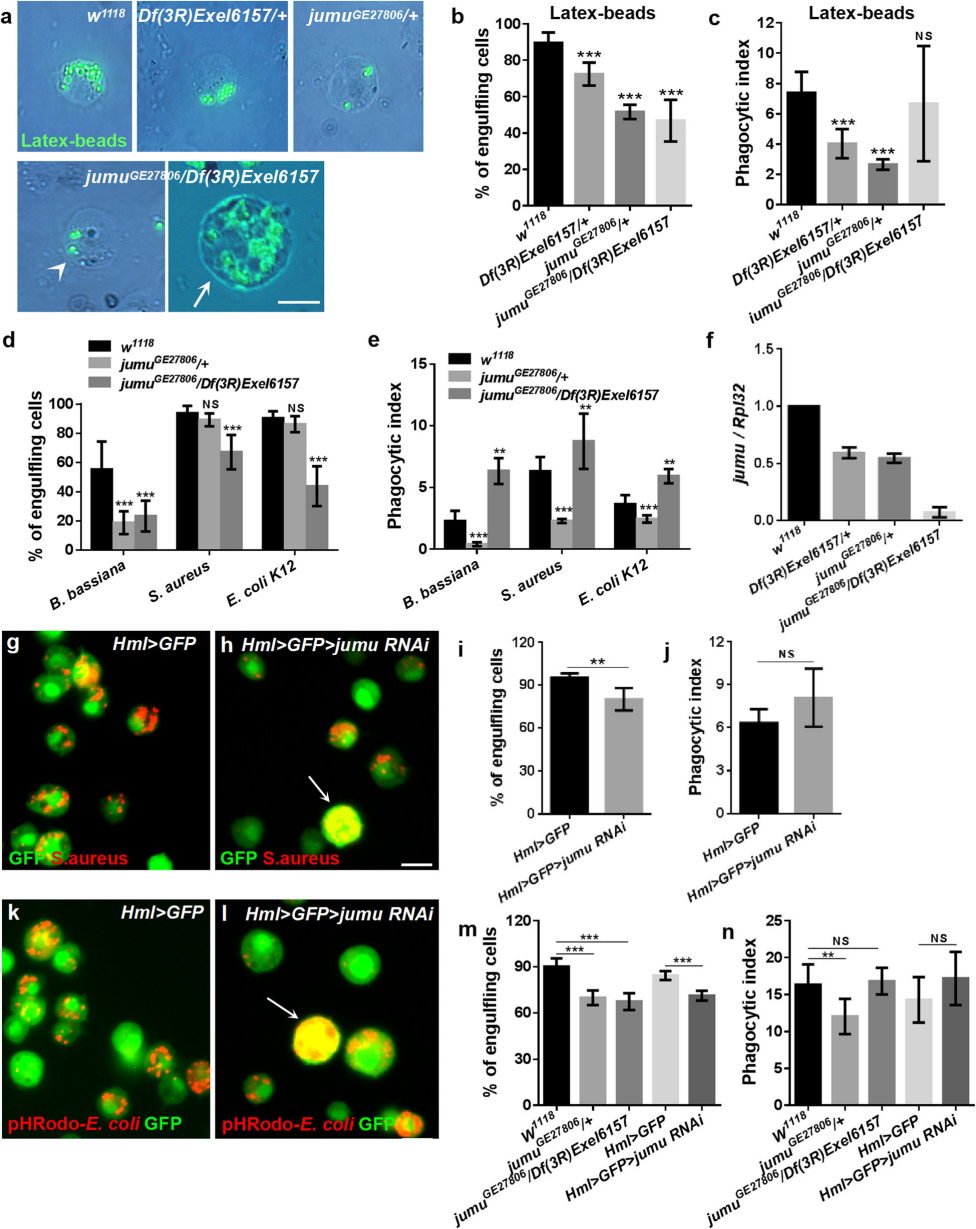
Loss of *jumu* results in defective phagocytosis in circulating hemocyte. **a, g, h, k, l** Circulating hemocytes were isolated from third-instar larvae 1 h after injection with latex beads, *B. bassiana*, *E. coli*, *S. aureus*at or pHRodo*-E. coli*. **b, d, i, m** Quantification of the percentage of engulfing cells in the phagocytosis assays of circulating hemocytes. **c, e, j, n** Quantification of phagocytosis indexes (the number of engulfed latex beads or bacteria per hemocyte) in phagocytosis assays of circulating hemocytes. **f** Real-time PCR analysis of the *jumu* level in the entire third-instar larvae. For all quantifications, the error bars represent ±S.E.M of at least 3 independent experiments; NS, not significant; **P < 0.01; ***P < 0.001 (Student’s *t*-test). The arrowhead in a indicates the *jumu*^*GE27806*^*/Df(3R)Exel6157* circulating hemocytes with normal size. The arrows in a, g and k indicate the enlarged circulating hemocytes resulting from the loss of *jumu*. Scale bars: 10 μm

In a previous study, we showed that heterozygous *jumu*^*GE27806*^ mutant adults (containing a P-element at the 5’ UTR of *jumu* in a forward orientation) exhibit defects in defenses against both fungi and bacteria [32]. To further investigate the role of Jumu in the phagocytosis of invading microbes by hemocytes, heterozygous *jumu*^*GE27806*^/+ and *Df(3R)Exel6157/+* (a small deficiency that deletes *jumu*) larvae were injected with fluorescent latex beads or pathogens. At 1 h postinfection, the circulating hemocytes were isolated, and the number of engulfing cells and phagocytosis indexes (i.e., the number of engulfed latex beads or bacteria per hemocyte) were determined. Nearly 90% of the hemocytes were able to engulf latex beads, with a phagocytosis index (PI) of 7.397 ± 0.4358 in *w*^*1118*^ third-instar larvae (Fig. 1a-c). However, the *jumu* heterozygous mutants showed a poor ability to engulf latex beads (Fig. 1a). Only 70 and 50% of the hemocytes were able to engulf latex beads, with PIs of 4.037 ± 0.3056 and 2.656 ± 0.1094, in *Df(3R)Exel6157/+* and *jumu*^*GE27806*^*/+*, respectively (Fig. 1b and c). Next, we further tested the phagocytosis ability of *jumu*^*GE27806*^*/+* mutants against *B. bassiana*, *S. aureus* or *E. coli*. Although the ratio of engulfing cells to total hemocytes was not obviously reduced during the phagocytosis of *S. aureus* and *E. coli*, the PIs for the three pathogens were reduced significantly in heterozygous *jumu*^*GE27806*^ compared with those in *w*^*1118*^ (Fig. 1d and e). Because all homozygous null *jumu* mutants died during embryogenesis, to achieve a more severe deficiency of *jumu* expression, we utilized double heterozygotes generated from crosses between *Df(3R)Exel6157/+* and *jumu*^*GE27806*^*/+*. In the analysis of the phagocytosis of the latex beads and pathogens, compared with *w*^*1118*^, the double heterozygote displayed an obvious reduction in the ratio of engulfing cells to the total number of circulating hemocytes (Fig. 1b and d). However, the PI for the phagocytosis of latex beads was not reduced, and the PIs for the phagocytosis of pathogens were increased in *jumu*^*GE27806*^*/Df(3R)Exel6157* (Fig. 1c and e). Similar to *jumu* heterozygous, most *jumu*^*GE27806*^*/Df(3R)Exel6157* hemocytes with a normal size also displayed a poor phagocytosis ability (Fig. 1a, arrowhead). However, we found that approximately 20% of hemocytes were greatly enlarged to 4–6 times the size of control cells, and most of these enlarged hemocytes (diameter > 20 μm) exhibited a strong ability to engulf latex beads and pathogens, leading to the increased PIs (Fig. 1a, arrow). These phenomena indicate that Jumu may affect the size of hemocytes in an autonomous and dose-dependent manner. To verify this hypothesis, the transcription levels of *jumu* in *jumu* mutant third-instar larvae were quantified by real-time PCR. The *jumu* mRNA levels were reduced by approximately 50% in the heterozygotes and nearly by 15-fold in the double heterozygote mutants (Fig. 1f). We next knocked down *jumu* using the hemocyte-specific driver *Hml-Gal4.* Knockdown of *jumu* at 29 °C led to reduced numbers of engulfing cells and the generation of enlarged cells exhibiting strong phagocytosis in circulating hemocytes (Fig. 1g-j, arrows). These results show that the enlarged cells resulting from the loss of *jumu* can effectively engulf invading pathogens and latex beads. Therefore, we next investigated whether these cells could digest the engulfed microbes. The acidification of mature phagosomes allows the digestion of engulfed particles; thus, we detected the maturation of phagosomes in *jumu* mutant hemocytes using *E. coli* labeled with pHrodo, which is a pH-sensitive dye that fluoresces in the acidic environment of a mature phagosome when it fuses with lysosomes. We found that the enlarged hemocytes could engulf the pHrodo-*E. coli* in an acidified mature phagosome (Fig. 1i, arrows), and the phagocytosis ability for pHrodo-*E. coli* was similar to the phagocytosis of FITC-*E. coli* in the hemocytes of *jumu* heterozygotes or *jumu* double heterozygotes (Fig. 1m and n)*.* Taken together, these results indicate that the loss of Jumu can decrease the uptake of pathogens and latex beads and disrupt the development of circulating hemocytes in a dose-dependent manner, but it does not affect the formation of mature phagosomes.

### Jumu regulates phagocytosis by modulating the expression of NimC1

**Fig. 2:**
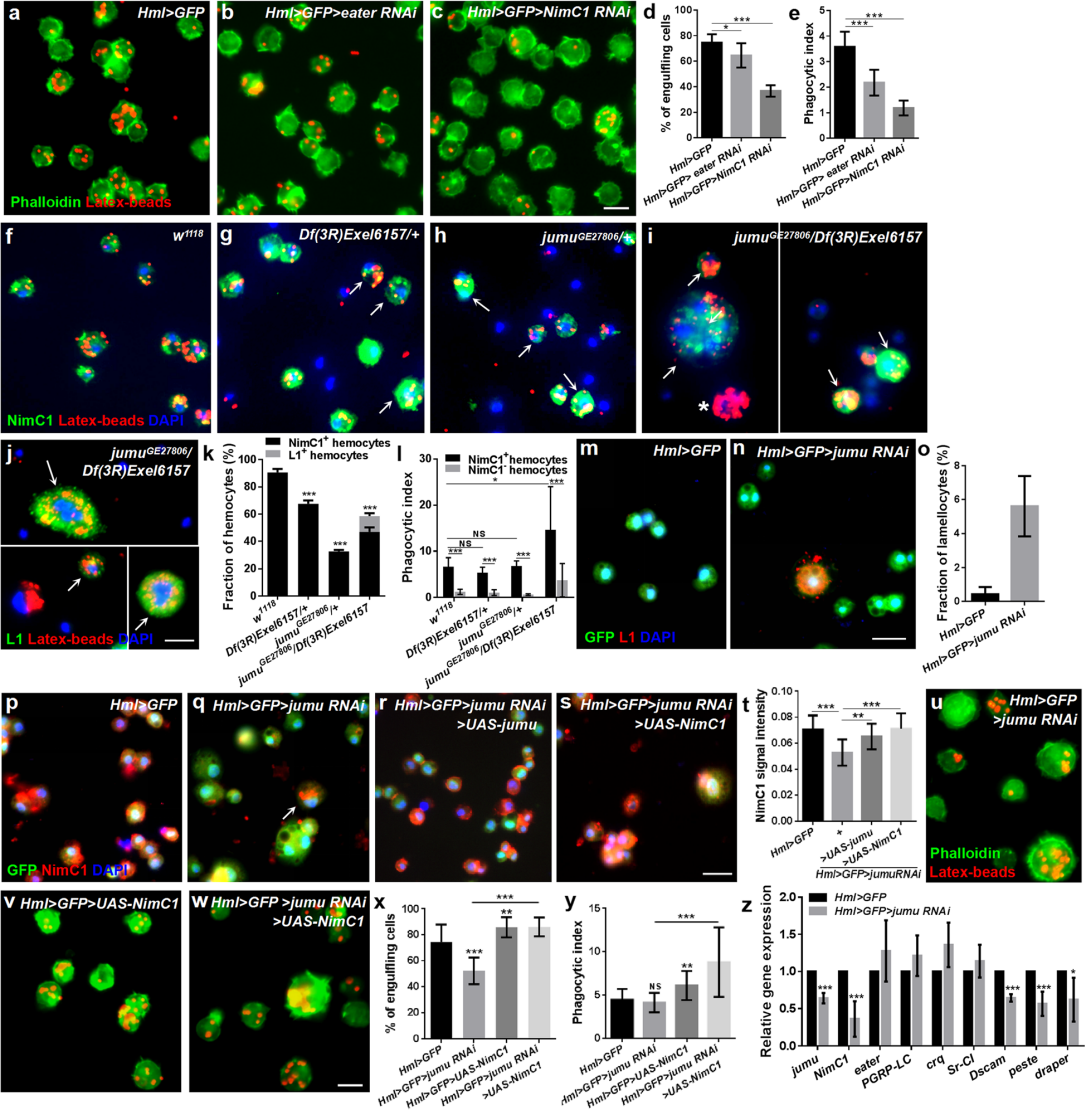
Jumu regulates hemocyte phagocytosis via affecting the expression of NimC1. **a-c** Hemocytes were isolated from third-instar larvae injected with latex beads (red) 1 h postinjection. The actin cytoskeleton was visualized using Alexa Fluor 488-labeled phalloidin (green). **d, e** Quantification of the percentage of engulfing cells and phagocytic indexes in phagocytosis assays. **f-i** Immunostaining against NimC1 (green) shows that most circulating hemocytes with strong phagocytosis ability are NimC1-positive in *jumu* heterozygotes (arrows) (g and h). However, in addition to NimC1-positive circulating hemocytes (arrows), nearly 10% of the NimC1-negative circulating hemocytes (asterisks) also display strong phagocytosis ability in *jumu* double heterozygotes (i). **j** Immunostaining against L1 (green) shows that lamellocytes also exhibit strong phagocytosis ability in *jumu* double heterozygotes (arrows). **k** Quantification of the percentage of NimC1-positive and L1-positive circulating hemocytes. **l** Quantification of phagocytosis indexes. **m-o** Immunostaining against L1 (red) shows that nearly 6% of the lamellocytes are observable in *Hml > GFP > jumu RNAi* circulating hemocytes. **p-s** Immunostaining against NimC1 (red) shows that NimC1 expression is reduced in *Hml > GFP > jumu RNAi* circulating hemocytes compared with that in the controls, and the overexpression of *jumu* and *NimC1* increases the NimC1 level in *Hml > GFP > jumu RNAi*. **t** Quantification of the NimC1 signal intensity in circulating hemocytes. **u-w** Hemocytes were isolated from third-instar larvae injected with latex beads (red) 1 h postinjection. The actin cytoskeleton was visualized using Alexa Fluor 488-labeled phalloidin (green). **x, y** Quantification of the percentage of engulfing cells and phagocytic indexes in phagocytosis assays. **z** Real-time PCR analysis of phagocytosis receptor genes levels of circulating hemocytes. Error bars represent the S.E.M of at least 3 independent experiments; not significant; *P < 0.05; **P < 0.01; ***P < 0.001 (Student’s *t*-test in d, e, k, o, z; one-way ANOVA in l, t, x, y). Scale bars: 10 μm (a-j, u-w); 20 μm (m-s)

The first step in phagocytosis is the recognition of microbes through a receptor on phagocytes. NimC1 and Eater have been suggested to be the phagocytosis receptors required for the phagocytosis of *E. coli* and *S. aureus* [13, 39], both of which are also markers of plasmatocytes [40]. In this study, we found that the knockdown of *Eater* and *NimC1* also reduced the phagocytosis of the latex beads (Fig. 2a-e). Thus, we first asked whether the loss of *jumu* causes a deficiency of NimC1 or Eater and affects the phagocytosis of hemocytes. We found that more than 90% of the *w*^*1118*^ circulating hemocytes show positive staining for NimC1 and display a strong capacity to engulf the latex beads, while 30 and 70% of the hemocytes could not be marked by NimC1 in *Df(3R)Exel6157/+* and *jumu*^*GE27806*^*/+,* respectively, and these hemocytes exhibited a reduced phagocytosis ability compared with that of the NimC1^+^ hemocytes (Fig. 2f-h, k and l; Additional file 2: Figure S1a-c). A previous study reported that multiple fly stocks and transgenic lines exhibit variations in NimC1 expression due to naturally occurring deletions and insertions at the NimC1 locus [41]. Thus, to ensure that the variations in the NimC1 expression levels in the *jumu* mutants were not attributed to this potential factor, we examined the expression of the *NimC1* gene using RT-PCR as previously described (variation of NimC1 expression in *Drosophila* stocks and transgenic strains) and found that only the appropriately sized products of the *NimC1-RA* and *NimC1-RB* genes are present in the *jumu* mutants (Additional file 2: Figure S1d). However, we found that all circulating hemocytes in the *jumu* mutants could be marked by Eater-GFP and an anti-H2 antibody (pan hemocyte marker) (Additional file 2: Figure S1e-j). Similar to the heterozygous *jumu* mutants, only approximately 50% of the *jumu*^*GE27806*^*/Df(3R)Exel6157* hemocytes could be marked by NimC1 and displayed a strong capacity to engulf the latex beads (Fig. 2i, arrow, k and l; Additional file 2: Figure S1 k). However, we found that some NimC1^−^ hemocytes also exhibited a strong phagocytosis ability, and in terms of their shape, some large hemocytes in *jumu*^*GE27806*^*/Df(3R)Exel6157* had the appearance of lamellocytes (Fig. 2i, asterisks). Thus, we next evaluated the lamellocytes of *jumu* mutants using an anti-L1 antibody. Few lamellocytes could be detected in *w*^*1118*^, *Df(3R)Exel6157/+* and *jumu*^*GE27806*^*/+*(data not shown)*.* However, we found that more than 10% of the hemocytes in *jumu*^*GE27806*^*/Df(3R)Exel6157* could be marked by L1, and most of these cells could effectively engulf the latex beads (Fig. 2j, arrow and k; Additional file 2: Figure S1 l, arrow). These results suggest that NimC1 levels affect the hemocyte-dependent phagocytosis of latex beads and that lamellocytes also have the ability to engulf latex beads. To further investigate whether *jumu* regulates the expression of NimC1 in plasmatocytes in a cell-autonomous manner, we detected the level of NimC1 in the hemocytes of *Hml > GFP > jumu RNAi* (*Hml-Gal4* begins to be expressed in second instar circulating cells), and *Gcm > jumu RNAi* (*Gcm-Gal4* begins to be expressed in embryonic circulating cells)*.* We detected multinucleate cells and lamellocytes among the circulating hemocytes (Fig. 2m-o; Additional file 2: Figure S1 m and n), and the immunostaining signal of NimC1 was reduced in *Hml > GFP > jumu RNAi* and *Gcm > jumu RNAi* compared with that observed in the control (Fig. 2p, q and t; Additional file 2: Figure S1o and p). The results of immunostaining against NimC1 and L1 showed that the round enlarged multinucleate cells caused by *jumu* deficiency are mainly plasmatocytes and lamellocytes (Fig. 2i, j and n; Additional file 2: Figure S1 k-p). We found that the overexpression of *jumu* can reduce the generation of multinucleate cells and lamellocytes and increase the expression of NimC1 in the hemocytes of *Hml > GFP > jumu RNAi* (Fig. 2r and t). Moreover, the overexpression of the *NimC1* gene could rescue the expression level of the NimC1 protein and phagocytosis but could not recuse the enlarged multinucleate cells in the hemocytes of *Hml > GFP > jumu RNAi* (Fig. 2s-y). We next detected transcription levels of the *NimC1* gene and the other seven receptor genes in circulating hemocytes. The knockdown of the *jumu* gene under the control of *Hml-Gal4* led to decreased transcription levels of *NimC1*, *Dscam*, *peste* and *draper* (Fig. 2z)*.* Similar to *Hml > GFP > jumu RNAi*, the *jumu* heterozygous hemocytes also displayed a reduced expression of these genes (Additional file 2: Figure S1q). It has been suggested that *Dscam*, *peste* and *draper* are phagocytic recognition receptors and required for microbial phagocytosis [39]. Thus, the decreased mRNA levels of these receptors might also strengthen the phagocytic defects in *jumu* mutants. Taken together, these results suggest that the loss of *jumu* can decrease the phagocytosis of hemocytes by reducing NimC1 protein levels, reduce the transcription levels of *Dscam*, *peste* and *draper* in circulating hemocytes and induce the generation of lamellocytes.

### Loss of *jumu* and *NimC1* in hemocytes cause defects of filopodia

**Fig. 3:**
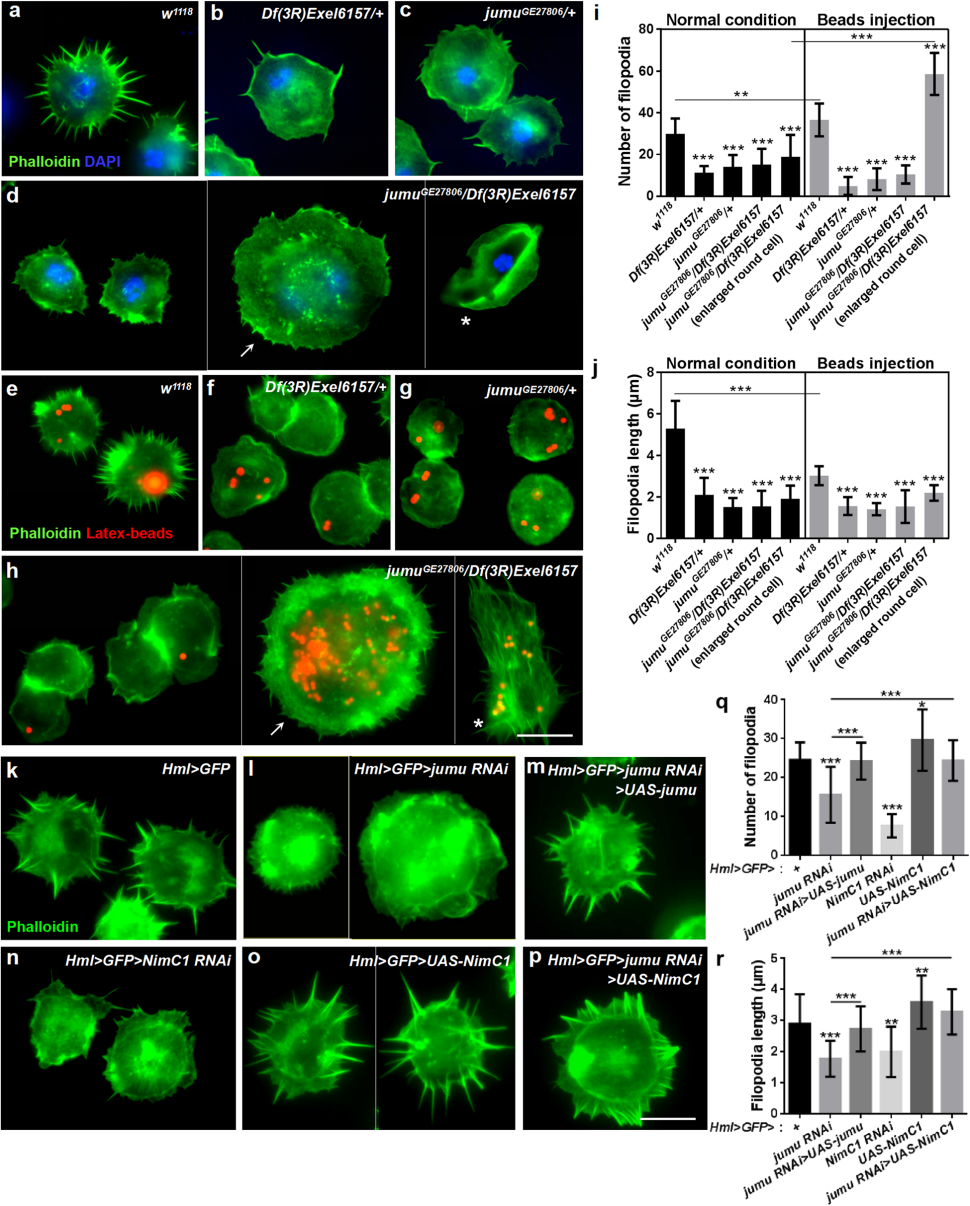
Loss of *jumu* or *NimC1* in circulating hemocytes causes filopodia defects. **a-d** Phalloidin staining (green) of circulating hemocytes shows a reduced number and length of filopodia in *jumu* mutant hemocytes compared with those in the controls. The arrow shown in D indicates an enlarged round hemocyte, and the asterisk shown in d indicates a lamellocyte. **e-h** Phalloidin staining (green) of circulating hemocytes isolated from third-instar larvae injected with latex beads (red). The *jumu* mutants show filopodium defects (**f-h**), although a portion of the enlarged round hemocytes (arrow) and lamellocytes (asterisk) exhibit an elongation of filopodia in the *jumu* double heterozygotes (**h**). **i, j** Quantification of the number (**i**) and length (**j**) of filopodia. **k-p** Phalloidin staining (green) of circulating hemocytes shows a reduced number and length of filopodia in *jumu* or *NimC1* knockdown hemocytes compared with those in the control (**l, n**), and the overexpression of *jumu* or *NimC1* can rescue the number and length of filopodia in the *jumu* knockdown hemocytes (**m, p**). **q, r** Quantifications of the number (**q**) and length (**r**) of filopodia. Error bars represent the S.E.M; * *P* < 0.05; **P < 0.01; ***P < 0.001 (one-way ANOVA). Scale bars: 10 μm

It has been suggested that phagocytosis also requires dynamic rearrangement of the plasma membrane, along with actin-dependent cytoskeletal remodeling, after activation of the signaling pathway by phagocytosis receptors [15, 42, 43]. Thus, we investigated whether the loss of *jumu* also affected the reorganization of the actin cytoskeleton and, consequently, phagocytosis. Phalloidin staining revealed numerous filopodia extending from the periphery of *w*^*1118*^ hemocytes (Fig. 3a). However, *jumu* heterozygous and *jumu*^*GE27806*^*/Df(3R)Exel6157* displayed obvious reductions in the number and length of filopodia but did not affect the formation of lamellipodia (Fig. 3b-d, i and j). We found that the number of filopodia increased, but their length was shorter in *w*^*1118*^ hemocytes after the phagocytosis of latex beads (Fig. 3e, i and j). However, the injection of latex beads did not induce an increase in the number or changes in the length of filopodia in the hemocytes of *jumu* mutants but did increase the number of filopodia in the enlarged round cells of the *jumu*^*GE27806*^*/Df(3R)Exel6157* mutant (Fig. 3f-j). In addition, we found that *jumu*^*GE27806*^*/Df(3R)Exel6157* lamellocytes exhibited large lamellipodia at the periphery under normal conditions, but after the phagocytosis of latex beads, the lamellocytes also extended numerous longer filopodia from the plasma membrane (Fig. 3d and h, asterisk). Similar to the *jumu* mutants, *jumu* RNAi under the control of *Hml-Gal4* also led to reduction in the number and length of filopodia in circulating hemocytes, and overexpressing *jumu* could recuse the phenotypes of filopodia observed in *Hml > GFP > jumu RNAi* (Fig. 3k -m, q and r). The above results suggested that loss of *jumu* likely decreases phagocytosis by reducing NimC1 expression. Therefore, we next asked whether the phagocytosis receptors also affected the formation of filopodia. Phalloidin staining showed that deficiency of *NimC1* also caused obvious reduction in filopodium formation (Fig. 3n, q and r). Moreover, the overexpression of *NimC1* increased the number and length of the filopodia in circulating hemocytes and rescued the phenotypes of the filopodia observed in *Hml > GFP > jumu RNAi* (Fig. 3o-r). Taken together, these results suggest that Jumu may maintain the normal filopodium formation by modulating the expression of NimC1.

### Jumu affects the expression of the proteins associated with the formation of actin filopodia

**Fig. 4:**
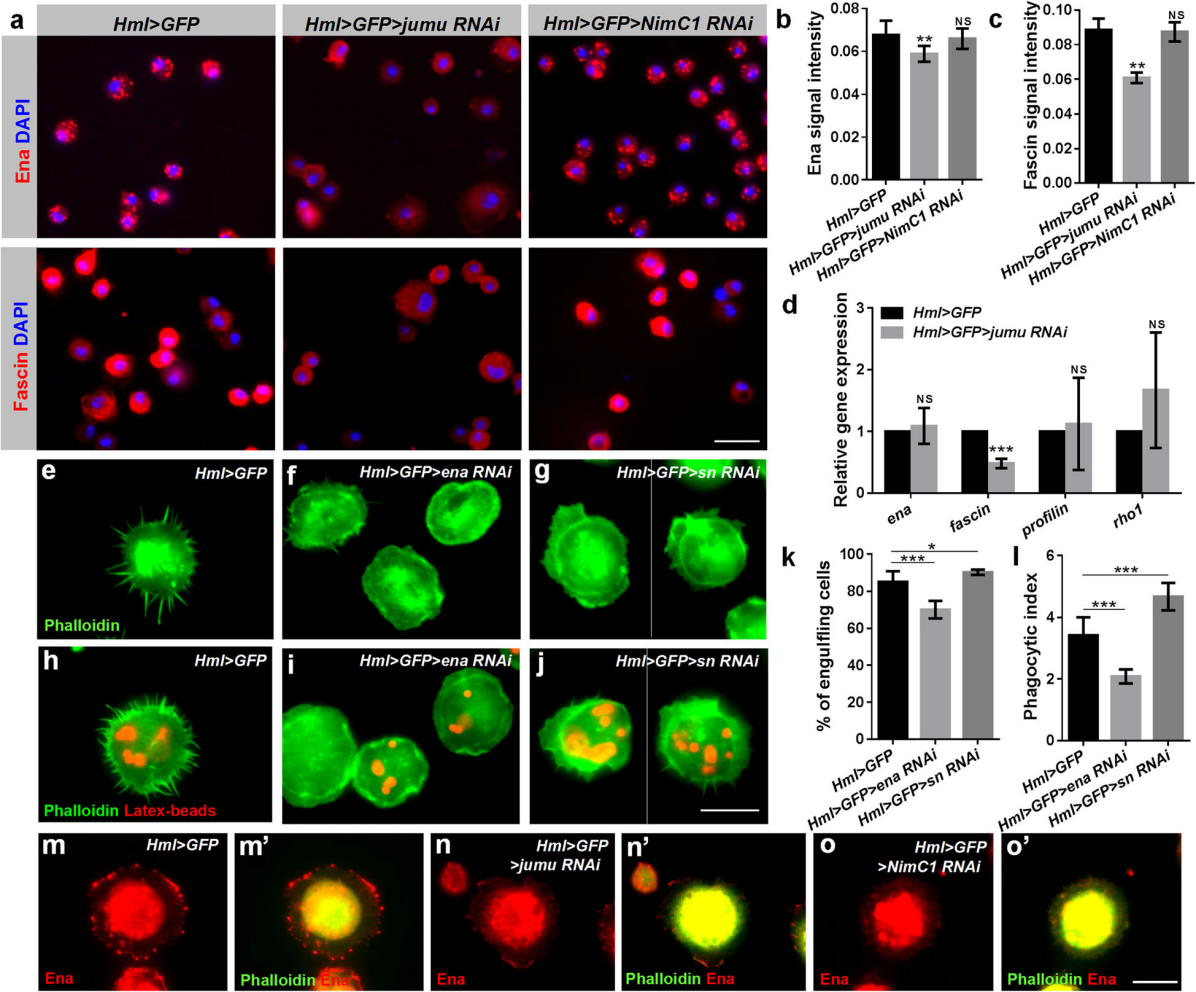
Expression levels of proteins associated with actin filopodium formation are changed in *jumu* knockdown hemocytes. **a** Immunostaining against Ena (red) and Fascin (red) shows that the expression of Ena and Fascin is reduced in *jumu* knockdown hemocytes compared with the expression in the controls; however, the knockdown of *NimC1* does not affect the expression of Ena and Fascin. **b, c** Quantification of signal intensities. **d** Real-time PCR analysis of *ena*, *fascin*, *profilin* and *rho1* levels in *jumu* knockdown hemocytes. **e-g** Phalloidin staining (green) shows that the number and length of filopodia are reduced in the circulating hemocytes of *Hml > GFP > ena RNAi* and *Hml > GFP > sn RNAi*. **h-j** Circulating hemocytes of *Hml > GFP > ena RNAi* and *Hml > GFP > sn RNAi* isolated from third-instar larvae injected with latex beads (red) 1 h postinjection show defects in filopodia (green). **k, l** Quantification of the percentage of engulfing cells and phagocytic indexes based on phagocytosis assays. **m-o’** Immunostaining against Ena (red) and phalloidin staining (green) shows that Ena is enriched at the tips of filopodia and lamellipodia in control circulating hemocytes; however, the expression level of Ena is markedly reduced at the tips of filopodia and lamellipodia in *Hml > GFP > jumu RNAi* (n and n’) and *Hml > GFP > NimC1 RNAi* (o and o’) circulating hemocytes. Error bars represent the S.E.M of at least 3 independent experiments; NS, not significant; *P < 0.05; **P < 0.01; ***P < 0.001 (Student’s t-test). Scale bars: 20 μm (a); 10 μm (e-j, m-o’)

A previous study suggested that Ena, Fascin, Rho1 and Profilin participate in the process of filopodium formation, and the loss of these factors can lead to defects in the number and length of filopodia [20, 24, 25, 44]. Thus, we asked whether the absence of filopodia in *jumu* mutants was related to these factors. We first determined the expression levels of Ena, Fascin, Rho1 and Profilin in hemocytes. We found that the Ena and Fascin signals were dramatically decreased, but the levels of Rho1 and Profilin were slightly increased in *jumu* mutants compared with those in *w*^*1118*^ (Additional file 2: Figure S2a-d). Similarly, the reduced Ena and Fascin signals were also shown in *Hml > GFP > jumu RNAi*, but the expression levels of Rho1 and Profilin remained unchanged (Fig. 4a-c; Additional file 2: Figure S3a-c). In addition, we knocked down *ena* and *fascin* in hemocytes and observed an absence or shortening of filopodia, and knockdown of *ena* in particular resulted in hemocytes that rarely extended spiky protrusions (Fig. 4e-j). The above results suggest that loss of *jumu* can cause a decrease in NimC1 levels, and loss of *NimC1* also leads to defective filopodia. Thus, we next asked whether *jumu* indirectly affects the expression of Ena and Fascin by regulating the expression of NimC1. However, we found that the expression levels of Ena, Fascin, Rho1 and Profilin proteins were not reduced in *NimC1* knockdown hemocytes (Fig. 4a-c; Additional file 2: Figure S3a-c). Next, we detected the transcription levels of *ena, fascin, rho1* and *profilin* and found that knockdown of *jumu* reduced the mRNA level of *fascin* (Fig. 4d). Moreover, we observed the subcellular localization of the four proteins. Immunostaining showed that loss of *jumu* did not markedly change the subcellular localization of Fascin, Rho1 and Profilin, except for a slight defect of Ena subcellular localization at the tips of filopodia and lamellipodia (Fig. 4m-n’; Additional file 2: Figure S2e-h). The Ena signal was primarily enriched at the tips of filopodia in control circulating hemocytes but was decreased at the tips of filopodia and lamellipodia in *jumu*-deficient hemocytes (Fig. 4 m-n’). Fascin and Rho1 localized to the lamellipodia and filopodia in control and *jumu* mutant hemocytes Additional file 2: Figure S2f and g). Profilin was distributed throughout the cell but was rarely observed at the leading edge of lamellipodia and filopodia (Additional file 2: Figure S2 h). Interestingly, we found that loss of *NimC1* also caused an obviously alteration in the subcellular localization of Ena at the tips of filopodia and lamellipodia (Fig. 4o and o’). This result demonstrates that the mechanism whereby Jumu regulates the levels of Ena and Fascin is independent of NimC1, but Jumu may affect the subcellular localization of Ena by regulating the expression of NimC1.

To further identify the relationship between cell spreading and phagocytosis, we evaluated the phagocytosis of latex beads after knockdown of *ena* and *fascin*. The phagocytosis ability was reduced in the *ena* knockdown hemocytes; however, compared with that in the control, the knockdown of *fascin* in the hemocytes increased the phagocytosis of latex beads (Fig. 4h-l). Similar to Fascin, a previous study showed that loss of *profilin* suppresses the formation of filipodia but also causes increased phagocytosis, and the authors suggested that the loss of *profilin* may change the balance between elongation and filament branching and lead to greater membrane ruffling, thereby increasing phagocytosis indirectly [44]. These results suggest that the proteins associated with the formation of actin filopodia regulate phagocytosis by different manners.

### Overexpression of *jumu* induces enhanced cell spreading and large numbers of filopodia

**Fig. 5:**
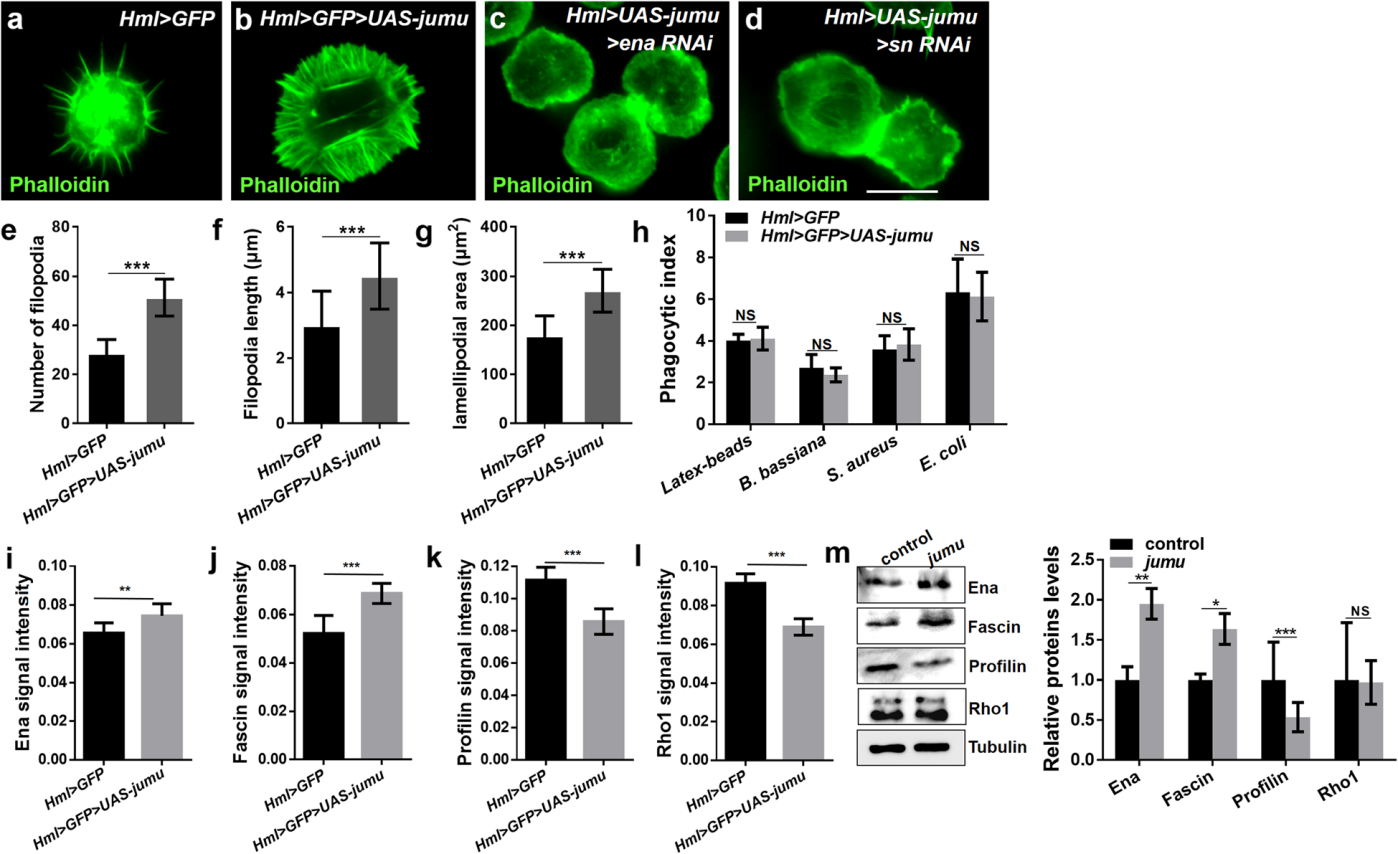
Overexpression of *jumu* induces cell spreading and large numbers of filopodia in hemocytes. **a-d** Phalloidin staining (green) shows that *Hml > GFP > UAS-jumu* displays an increased number of filopodia and larger lamellipodia than the control (**a** and **b**); knockdown of *ena* or *fascin* in *Hml > GFP > UAS-jumu* can inhibit cell spreading and the formation of numerous filopodia (**c** and **d**). **e-g** Quantification of filopodium numbers and lengths and lamellipodial area. **h** Quantification of the phagocytosis indexes for latex beads, *B. bassiana*, *S. aureus* or *E. coli.* **i-l** Quantification of Ena, Fascin, Rho1 and Profilin levels. **m** Western blot analysis using antibodies against Ena, Fascin, Profilin and Rho1 and the corresponding graphical representation show that the levels of Ena and Fascin are increased in *jumu*-overexpressing S2 cells compared with the expression in the controls, whereas the levels of Profilin are reduced and the levels of Rho1 are unchanged. Error bars represent the S.E.M of at least 3 independent experiments; NS, not significant; *P < 0.05; **P < 0.01; ***P < 0.001 (Student’s *t-*test). Scale bars: 10 μm

Next, we asked whether Jumu would be sufficient to induce the formation of lamellipodia and filopodia. As expected, numerous filopodia extended radially throughout the lamellipodia, and the number and length of filopodia as well as the area of the lamellipodia were obviously increased in hemocytes overexpressing *jumu* (Fig. 5a, b, e-g). However, despite inducing an increased lamellipodia area and filopodia number, the overexpression of *jumu* did not sufficiently enhance the phagocytosis ability of hemocytes. The PIs for the latex beads and pathogens did not differ in *Hml > GFP > UAS-jumu* compared with those in the control (Fig. 5h). First, we determined whether the overexpression of *jumu* induces numerous filopodia by increasing the expression of NimC1. However, although the loss of *jumu* reduced the expression of NimC1, the overexpression of *jumu* is insufficient to increase the expression level of NimC1 (Additional file 2: Figure S4a-d). Next, we detected the expression of Ena, Fascin, Rho1 and Profilin and found that in contrast to the loss of *jumu*, the overexpression of *jumu* caused an increase in the expression levels of Ena and Fascin and a decrease in the expression levels of Profilin and Rho1 (Fig. 5i-l). To further evaluate the role of *jumu* in the regulation of Ena, Fascin, Rho1 and Profilin, we transfected the full-length *jumu* gene into S2 cells and then detected the expression levels of these four proteins through Western blotting. Similar to the in vivo experiments, compared with the control cells, the overexpression of *jumu* in the S2 cells caused increases of 90 and 60% in the protein levels of Ena and Fascin, respectively, and a 50% reduction in the Profilin expression level; however, the Rho1 expression level was not reduced (Fig. 5m). The overexpression of *jumu* did not affect the subcellular localization of Ena, Fascin, Profilin and Rho1, but an enhanced enrichment of Ena was observed at edges of the lamellipodia and filopodia (Additional file 2: Figure S4e). To further examine whether the increases in lamellipodia and filopodia in *jumu-*overexpressing hemocytes were caused by the increases in Ena and Fascin, we knocked down *ena* or *fascin* in *Hml > GFP > UAS-jumu* hemocytes. Phalloidin staining showed that knockdown of *ena* or *fascin* efficiently inhibited the elongation of lamellipodia and filopodia in *Hml > GFP > UAS-jumu* hemocytes (Fig. 5c and d). Taken together, these results indicated that Jumu is sufficient to promote the formation of lamellipodia and filopodia by increasing the expression levels of Ena and Fascin.

### Jumu maintains proper hemocyte division by regulating the cell cycle and cytokinesis

**Fig. 6:**
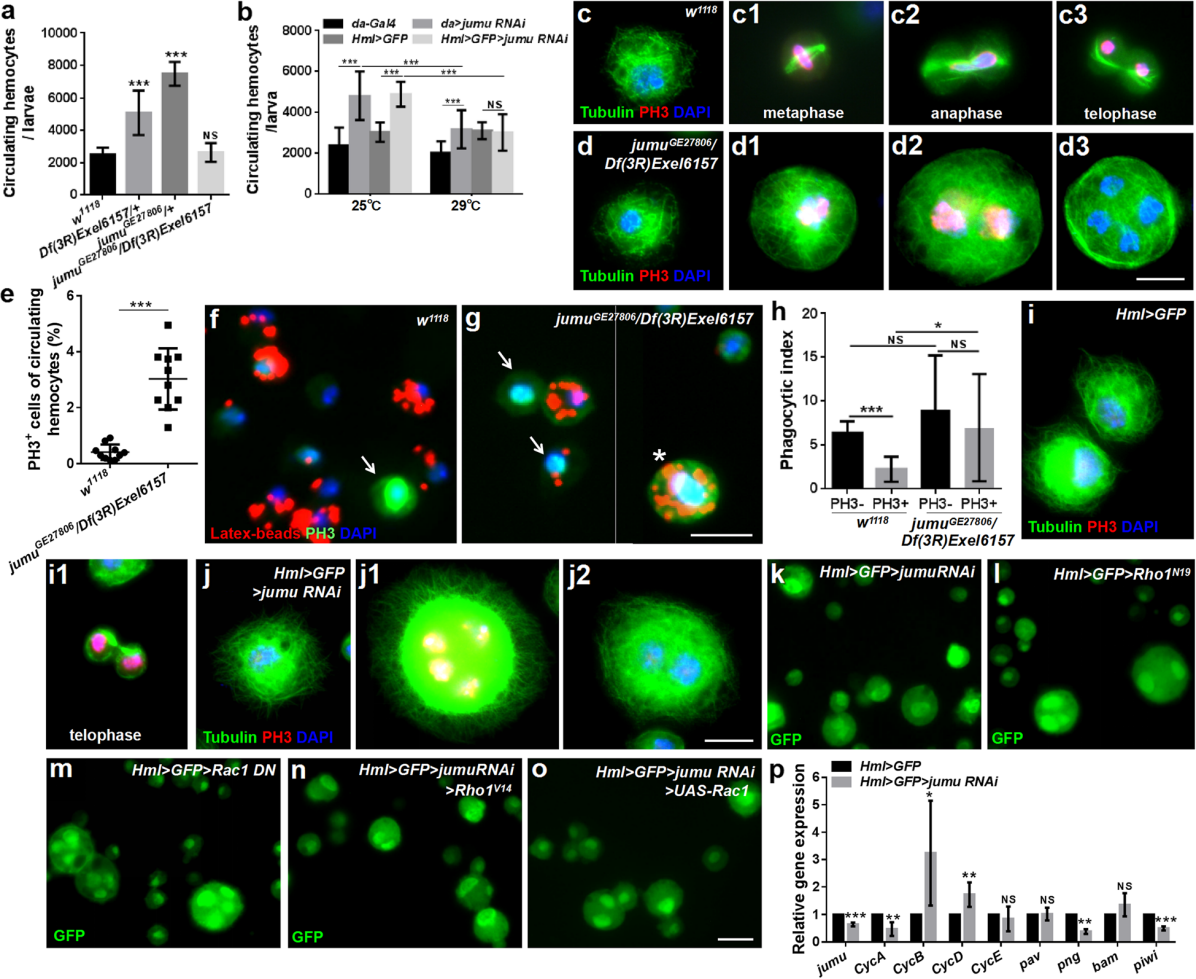
Severe deficiency of *jumu* impairs hemocyte division by affecting the cell cycle and cytokinesis. **a** Quantification of circulating hemocyte numbers in *jumu* mutant third-instar larvae. **b** Quantification of circulating hemocyte numbers in third-instar larvae of *da > jumu RNAi* and *Hml > GFP > jumu RNAi* (*da >* ^*w1118*^*, da > jumu RNAi*, *Hml > GFP >* ^*w1118*^ and *Hml > GFP > jumu RNAi* were raised at 25 °C or 29 °C). **c-d3** Immunostaining against Tubulin (green) and PH3 (red) shows that the circulating hemocytes that are not undergoing mitosis in *jumu* double heterozygotes display similar microtubule cytoskeletons to controls (c and d); the circulating hemocytes of controls in metaphase (c1), anaphase (c2), and telophase (c3) display clear spindles and cytokinesis. However, most PH3-positive circulating hemocytes in *jumu* double heterozygotes show defects in their spindles and cytokinesis (d1 and d2), and no cytokinesis is detectable in cells with divided nuclei (d3). **e** Quantification of the percentage of PH3-positive cells in third-instar larval circulating hemocytes. **f, g** PH3 staining (green) of circulating hemocytes isolated from third-instar larvae injected with latex beads (red). **h** Quantification of phagocytic indexes of latex beads. **i-j2** Immunostaining against Tubulin (green) and PH3 (red) shows that *jumu* knockdown hemocytes display similar mitosis defects to those in *jumu* double heterozygotes hemocytes. **k-o** Expression of constitutively active Rho1 (*Rho1*^*V14*^) or overexpression of *Rac1* cannot rescue the enlarged cell size of *Hml > jumu RNAi* circulating hemocytes (n, o). **p** Real-time PCR analysis of genes associated with cell cycle and division levels of *jumu* knockdown hemocytes. For all quantifications: error bars represent the S.E.M; NS, not significant; *P < 0.05; **P < 0.01; ***P < 0.001 (Student’s *t*-test in a, e, p; one-way ANOVA in b, p). Scale bars: 10 μm (c-d3, i-j2), 20 μm (f, g, k-o)

We detected enlarged and multinucleate cells among the circulating hemocytes in *jumu*^*GE27806*^*/Df(3R)Exel6157* and *Hml > GFP > jumu RNAi* (Figs. 1a and 2n). In addition, a previous study showed that heterozygous *jumu*^*GE27806*^*/+* larvae display an increase in the number of circulating hemocytes [32]. Thus, we detected the number of circulating hemocytes in *jumu*^*GE27806*^*/Df(3R)Exel6157* third-instar larvae. However, we found that the number of circulating hemocytes was not increased in *jumu*^*GE27806*^*/Df(3R)Exel6157* larvae (Fig. 6a)*.* We speculated that this phenotype may be associated with enlarged multinucleate hemocytes in *jumu*^*GE27806*^*/Df(3R)Exel6157.* These phenomena indicate that Jumu may affect the number and size of hemocytes in a dose-dependent manner. To further verify this possibility, we next knocked down *jumu* using the ubiquitous driver *da-Gal4* or the hemocyte-specific driver *Hml-Gal4* under different temperatures to control the levels of *jumu* and subsequently determined the number of hemocytes. RNAi knockdown of *jumu* at 25 °C led to an increase in the number of hemocytes compared with the control; however, the severe deficiency of *jumu* caused by knockdown at 29 °C led to reduced numbers of hemocytes compared with the number of hemocytes observed following *jumu* RNAi at 25 °C, similar to the observation in the *jumu* double heterozygotes (Fig. 6b).

It has been suggested that a defect in cytokinesis or DNA overreplication can lead to enlarged cells [19, 45]. Moreover, the above results showed that the loss of *jumu* affects actin-dependent cytoskeletal remodeling; therefore, we speculate that the loss of *jumu* might also cause a defect in microtubule cytoskeleton rearrangement during mitosis. To investigate whether the enlarged hemocytes caused by the loss of *jumu* are due to these causes, third-instar larvae hemocytes were stained with antibodies against Tubulin and phospho-histone H3 (PH3) to visualize microtubules and cell mitosis. The hemocytes that were not undergoing mitosis showed a similar microtubule cytoskeletal organization in *w*^*1118*^ and *jumu*^*GE27806*^*/Df(3R)Exel6157* (Fig. 6c and d). We found that less than 1% of circulating *w*^*1118*^ hemocytes were undergoing mitosis (PH3^+^ cells), most of which displayed clear spindles, especially during metaphase, and nuclear division accompanied cytokinesis during anaphase and telophase (Fig. 6c1-c3 and e). However, more than 3% of circulating *jumu*^*GE27806*^*/Df(3R)Exel6157* hemocytes were PH3^+^ cells, nearly half of which did not display spindles or show signs of nuclear division associated with cytokinesis, and most defective cells were larger and multinucleated (Fig. 6d1-e). Next, we examined whether the phagocytic deficit observed in *jumu* lacking hemocytes is a secondary consequence of the defects in mitosis. We found that compared with the PH3-negative cells in *w*^*1118*^, the PH3-positive cells have an obviously reduced phagocytosis ability (Fig. 6f, h). However, the phagocytosis ability of the PH3-positive cells is not reduced in *jumu*^*GE27806*^*/Df(3R)Exel6157*, although some normally sized PH3-positive cells showed a reduced phagocytosis of latex beads, and the enlarged PH3-positive cells have a stronger phagocytosis ability (Fig. 6g, h). This result suggests that the phagocytic deficit of the hemocytes in *jumu*^*GE27806*^*/Df(3R)Exel6157* is not attributed to a mitotic deficit. Moreover, the *Hml > GFP > jumu RNAi* hemocytes displayed a mitotic phenotype similar to that observed in *jumu*^*GE27806*^*/Df(3R)Exel6157* (Fig. 6i-j2). We investigated whether the loss of *jumu* could also cause DNA overreplication in hemocytes. To investigate this possibility, we detected cells in the S phase through BrdU incorporation assays. However, the incorporation of BrdU was not increased in the *jumu*^*GE27806*^*/Df(3R)Exel6157* third-instar larvae hemocytes (Additional file 2: Figure S5a-c), and we found that the BrdU-positive cells and BrdU-negative cells in *w*^*1118*^ and *jumu*^*GE27806*^*/Df(3R)Exel6157* hemocytes had a similar phagocytosis ability (Additional file 2: Figure S5d-f’)*.* Moreover, the TUNEL staining showed that the *jumu*^*GE27806*^*/Df(3R)Exel6157* circulating hemocytes did not display an increase in apoptotic cells compared with the number in *w*^*1118*^ (Additional file 2: Figure S5 g and h). Taken together, these results suggest that compared with *jumu* heterozygotes, a severe deficiency in Jumu levels can induce hemocyte mitosis but inhibit the formation of spindles and cytokinesis, leading to the generation of enlarged hemocytes with multiple nuclei and a reduced number of circulating hemocytes in *jumu*^*GE27806*^*/Df(3R)Exel6157*.

Similar to cells lacking *jumu*, hemocytes expressing dominant-negative *Rho1* (*Rho1*^*N19*^) or *Rac1* (*Rac1 DN*) were enlarged and multinucleated (Fig. 6l and m) [19]. We investigated whether the enlargement of hemocytes resulting from the loss of *jumu* was related to the inactivation of Rho1 and Rac1. However, the expression of constitutively active *Rho1* (*Rho1*^*V14*^) and *Rac1* did not rescue the enlarged cell size of the *Hml > jumu RNAi* hemocytes (Fig. 6n and o), suggesting that *jumu* regulates hemocyte size in a Rho1- and Rac1-independent manner.

The above result shows that the loss of *jumu* causes an increased number of circulating hemocytes in the M phase and accelerates the cell cycle process. Thus, we investigated whether Jumu deficiency affects the expression of *Cyclins*. We detected the mRNA levels of *CycA*, *CycB*, *CycD* and *CycE* using real-time PCR and found that the knockdown of *jumu* reduces the *CycA* level and increases the *CycB* and *CycD* levels (Fig. 6p). Previously, we analyzed the gene expression profiles of larval circulating hemocytes with overexpression of *jumu* using the GeneChip Drosophila Genome 2.0 Array and found four genes, *pav*, *png*, *bam* and *piwi*, which were significantly upregulated (> 5-fold), participated in cell cycle and cell division according to gene ontology analysis (unpublished data). Moreover, a previous study showed that the RNAi of *pav* in the S2R or Kc cell could result in enlarged and multinucleate cells [46]*.* Therefore, we next detected the expression of *pav*, *png*, *bam* and *piwi* in *jumu*-deficient hemocytes. Quantitative RT-PCR indicated that the expression of *pav* and *bam* were not changed, but the transcription levels of *png* and *piwi* were significantly downregulated (Fig. 6p). Moreover, a similar change in the mRNA level of these genes was observed in the *jumu* mutant (Additional file 2: Figure S5i). These findings suggest that Jumu may control the cell cycle and mitosis process by affecting the expression of *Cyclin* genes, *png* and *piwi*.

### Knockdown of *jumu* induces the generation of lamellocytes via activation of the toll pathway in hemocytes

**Fig. 7:**
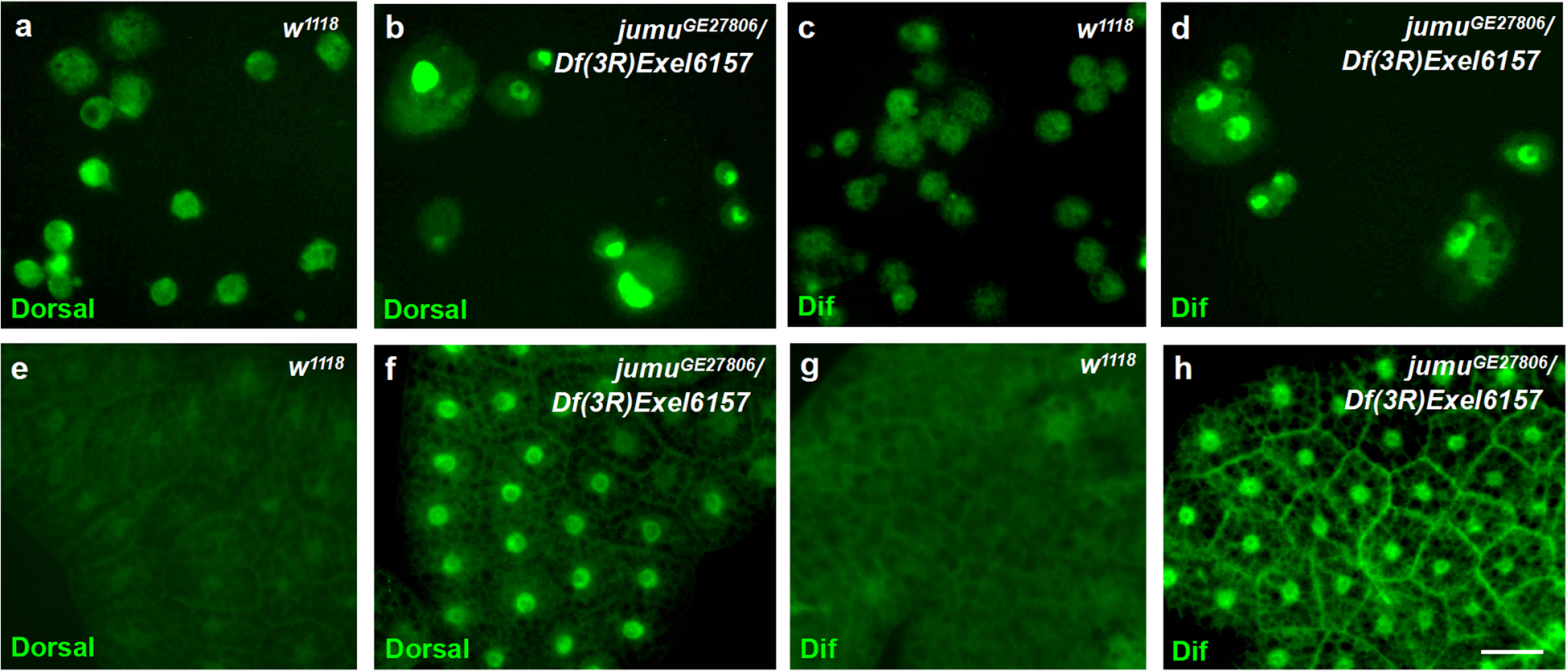
Severe deficiency of *jumu* leads to the activation of the Toll pathway. **a-h** Immunostaining against Dorsal (green) and Dif (green) shows that Dorsal/Dif are undetectable in the nuclei of control circulating hemocytes (**a**, **c**) and the fat body (**e**, **g**). However, most circulating hemocytes and the fat body exhibit nuclear localization of Dorsal/Dif in the *jumu* double heterozygotes (**b**, **d**, **f** and **h**). Scale bars: 10 μm (**a**- **d**); 50 μm (**e**-**h**)

**Fig. 8:**
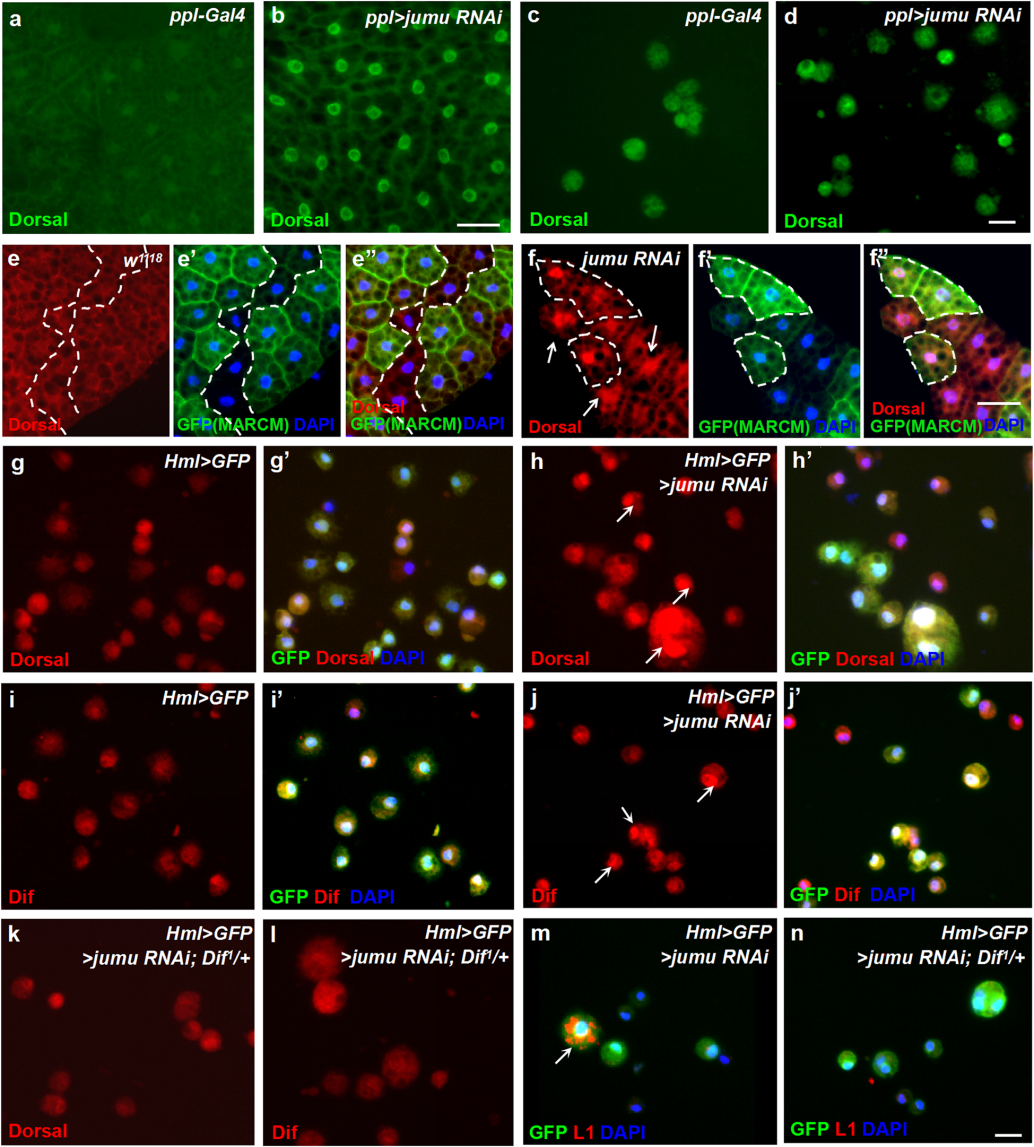
Knockdown of *jumu* in the fat body and hemocytes induces activation of the Toll pathway**. a-d** Immunostaining against Dorsal (green) shows that knocking down *jumu* in the fat body under the control of *ppl-Gal4* causes the nuclear localization of Dorsal in the fat body but not in circulating hemocytes. **e-f”** Clones expressing *jumu RNAi* (GFP^+^) also cause increased nuclear enrichment of Dorsal in adjacent wild-type clones (GFP^−^) (f-f”). **g-l** Immunostaining against Dorsal (red) and Dif (red) shows that the circulating hemocytes of *Hml > GFP > jumu RNAi* display activated Dorsal/Dif in the nuclei (arrows) (h and j), and *Dif* deficiency can rescue the nuclear localization phenotype of Dorsal/Dif (k and l). **m, n** Immunostaining against L1 (red) shows that *Dif* deficiency can reduce the generation of lamellocytes (arrows) in *Hml > GFP > jumu RNAi* circulating hemocytes. Scale bars: 20 μm (a, b, e-f”); 10 μm (c, d, g-n)

A previous study suggested that activation of the Toll pathway in hemocytes or fat body can induce lamellocyte formation [47]. Moreover, in a recent study, we demonstrated that loss of *jumu* in the entire lymph gland leads to the generation of lamellocytes through activation of Dif [34]. Thus, we next questioned whether the generation of lamellocytes in the circulating hemocytes of *jumu*^*GE27806*^*/Df(3R)Exel6157* was related to the Toll pathway. We found that Dorsal and Dif rarely exhibited nuclear localization in hemocytes and the fat body of *w*^*1118*^ but were obviously enriched in the nuclei of most of the *jumu*^*GE27806*^*/Df(3R)Exel6157* hemocytes and the fat body (Fig. 7). Next, to further investigate whether Jumu tissue autonomously participates in the activation of the Toll pathway in hemocytes and the fat body, we knocked down *jumu* in the fat body and hemocytes using *ppl-Gal4* and *Hml-Gal4*, respectively. The RNAi knockdown of *jumu* only in the fat body induced the activation of Dorsal in the fat body but did not cause the activation of Dorsal in circulating hemocytes or the generation of lamellocytes (Fig. 8a-d). This result suggests that lamellocyte formation caused by loss of *jumu* does not depend on activation of Toll signaling in the fat body. To further investigate whether *jumu* cells autonomously control the activation of Dorsal in the fat body, we used the MARCM technique to analyze the localization of Dorsal in GFP-marked clones. The clones expressing *jumu RNAi* (GFP^+^) did not show an increased nuclear enrichment of Dorsal compared with that in the wild-type clones (GFP^−^), and some wild-type clones showed activation of Dorsal (Fig. 8e-f”), suggesting that *jumu* noncell-autonomously affects the activation of the Toll pathway in the fat body. Moreover, the hemocytes of *Hml > GFP > jumu RNAi* exhibited nuclear enrichment of Dorsal and Dif (Fig. 8g-j’). Moreover, loss of *Dif* effectively inhibited the nuclear enrichment of Dorsal and Dif and reduced the generation of lamellocytes but did not rescue the enlarged cell phenotype in *Hml > GFP > jumu RNAi* (Fig. 8k-m’)*.* In addition to the Toll pathway, Janus Kinase/signal transducer and activator of transcription (JAK/STAT) and c-Jun N-terminal kinase (JNK) pathway activation in hemocytes also promote lamellocyte formation [48]. Thus, we next detected activation of the JAK/STAT and JNK pathways in *jumu*-deficient hemocytes by assessing the expression of target genes. However, we found that the transcription levels of JAK/STAT target genes *hop* and *Stat92E* and JNK target genes *puc* and *bsk* were not increased in *jumu*-deficient hemocytes (Additional file 2: Figure S6). Taken together, these results indicate that severe deficiency of *jumu* induces the generation of lamellocytes through activation of the Toll pathway in circulating hemocytes.

## Discussion

### Jumu regulates hemocyte phagocytosis by affecting NimC1 expression and cytoskeletal reorganization

**Fig. 9:**
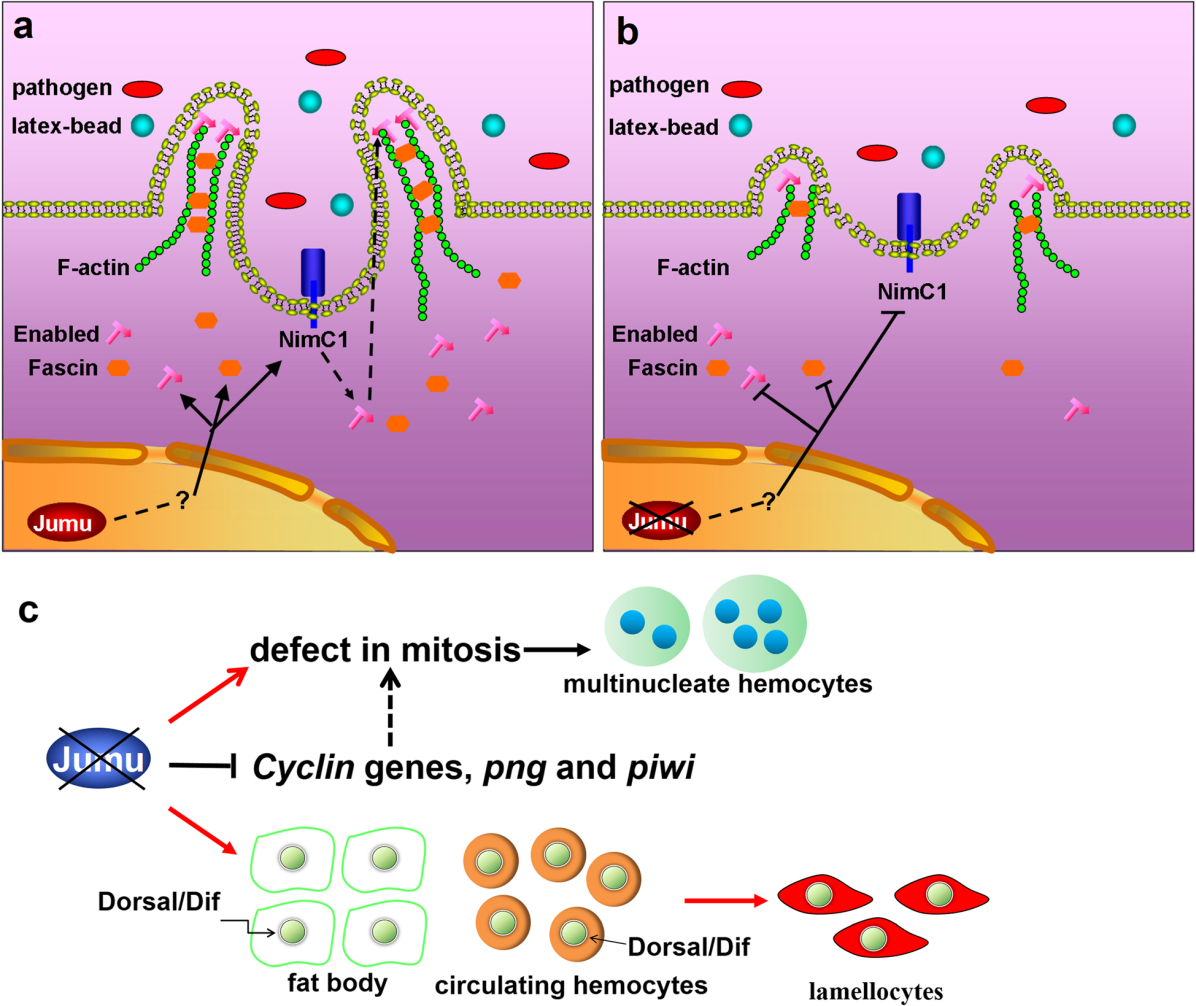
Model of the functions of Jumu in filopodium formation and circulating hemocyte phagocytosis. **a** Normal expression of Jumu maintains the levels of NimC1, Enabled and Fascin in a direct or indirect manner. Enabled and Fascin participate in filopodium formation. The phagocytosis receptor NimC1 is involved in the recognition of pathogens and the subcellular localization of Ena. **b** Deficiency of Jumu reduces the protein levels of NimC1, Enabled and Fascin and consequently inhibits filopodium formation and hemocyte phagocytosis. **c** Severe deficiency of *jumu* can inhibit normal hemocyte mitosis and result in enlarged multinucleated hemocytes. The loss of *jumu* may control the cell cycle and mitosis process by affecting the expression of the *Cyclin* genes *png* and *piwi*. Severe deficiency of *jumu* also induces the generation of lamellocytes through the activation of the Toll signaling pathway

*Drosophila* has become an excellent model for studies on phagocytic cell function during development and the elimination of pathogens [49]. Phagocytosis plays a central role in cellular immune responses to infection, which suggests that a better understanding of phagocytosis will lead to a better understanding of cellular immunity. A previous study suggested that *jumu,* which is an FKH transcription factor, is required for defense against fungal and Gram-positive bacterial infection; *jumu*-defective adult mutant flies were found to exhibit reduced survival and clear phagocytic defects after *B. bassiana* and *S. aureus* infection [32]. In the present study, we further confirmed the role of *jumu* in phagocytosis in larval circulating hemocytes. Loss of *jumu* caused generally defective phagocytosis of latex beads and pathogens by reducing NimC1 expression and affecting cytoskeletal reorganization (Fig. 9a and b). It has been suggested that NimC1 protein is required for the recognition of both *E. coli* and *S. aureus*. Similarly to Eater protein, NimC1 might directly bind to bacteria via its extracellular EGF-like repeats, or might indirectly act as a coreceptor at a later stage in the phagocytic process [13, 39]. In the present study, we found that NimC1 is also involved in the phagocytosis of latex beads. A general understanding is that phagocytes uptake beads in a manner not dependent on specific receptors. Therefore, NimC1 may regulate dynamic actin filament rearrangement via affecting the location of Enabled, then indirectly impact the engulfment process of latex-beads. The mechanism of latex bead phagocytosis has not yet been elucidated in *Drosophila*, and the contribution of NimC1 to latex bead phagocytosis will continue to be uncovered in our further studies.

In addition to microbial recognition by several receptors, the phagocytosis process requires actin filament rearrangement to engulf invading microbes. Some cytoskeletal regulatory proteins have been shown to regulate phagocytosis in *Drosophila*; for example, positive regulation occurs through SCAR and Arp2/3, whereas Profilin negatively regulates the phagocytosis of *E. coli* and *S. aureus* [44]. Additionally, Rho GTPases, such as Cdc42, Rac1 and Rac2, regulate phagocytosis by altering hemocyte cell shape after infection [42, 50, 51]. In the present study, we found that proteins associated with filopodium formation (Ena, Fascin and Rho1) also affect phagocytosis in hemocytes. Knockdown of *ena* led to a defect in the phagocytosis of latex beads, whereas loss of *fascin* increased the ability of the cells to engulf latex beads. *Ena*-deficient hemocytes rarely exhibited filopodia or extended lamellipodia under normal conditions or after latex bead infection, suggesting that Ena is involved in the regulation of plasma membrane protrusion during phagocytosis. Although the number of filopodia was reduced, numerous irregular lamellipodia were observed in *fascin-*deficient hemocytes after latex bead infection, which suggested that irregular lamellipodia may favor the dynamic rearrangement of the plasma membrane and that loss of *fascin* may promote cytoskeletal reorganization during phagocytosis. Additionally, a previous study indicated that the small GTPase Rho1 also positively controls the formation of cell protrusions [20]. However, we found that the knockdown of *Rho1* did not impair the phagocytosis ability of hemocytes (data not shown), the numbers of engulfing cells were not reduced, and the PIs of the latex beads and pHRodo*-E. coli* phagocytosis were increased. In fact, most *Rho1*-defective hemocytes showed similar phagocytosis to the controls, but the enlarged *Rho1*-defective hemocytes exhibited strong phagocytosis, which led to increased phagocytosis indexes (data not shown). Taken together, these results suggest that the proteins regulating the actin cytoskeleton affect phagocytosis in different manners, and the essential element for phagocytosis is dynamic actin filament rearrangement rather than simple filopodium elongation. Thus, the various regulations of cytoskeletal regulatory proteins on phagocytosis might explain why the increased lamellipodia area and filopodia number obtained with the overexpression of *jumu* did not sufficiently enhance the phagocytosis ability of hemocytes. Additionally, we also revealed that the loss of NimC1 affects the recruitment of Ena at the tips of filopodia and lamellipodia, and Jumu likely regulates the subcellular localization of Ena by altering the expression of NimC1 (Fig. 9a and b). The role of phagocytic receptors in the regulation of plasma membrane protrusion during phagocytosis is not well understood and has yet to be investigated.

### Loss of Jumu changes hemocyte differentiation

Here, we showed that Jumu affects the differentiation and development of circulating hemocytes. The activation of several signaling pathways, such as the JNK, JAK/STAT and Toll pathways, in circulating hemocytes can promote lamellocyte formation [47, 48]. In a previous study, we showed that overexpression of *jumu* in both the fat body and hemocytes induces melanotic nodules and lamellocytes by activating Toll signaling, but activated Dorsal/Dif were only found in hemocytes deposited on the fat body and aggregated circulating hemocytes, and not within the fat body or scattered circulating hemocytes [33]. In the present study, we further indicated that severe deficiency of *jumu* can autonomously induce activation of the Toll signal pathway in the fat body and circulating hemocytes, but lamellocyte formation caused by loss of *jumu* only depends on Toll signaling from circulating hemocytes (Fig. 9c). Moreover, in contrast to overexpression of *jumu*, loss of *jumu* in both the fat body and hemocytes does not induce melanotic nodules and deposited hemocytes. These results suggest that overexpression of *jumu* or a lack of *jumu* affects the activation of Toll signaling in a different manner, and the correct genetic dose of *jumu* balances the activation of immune signaling and prevents chronic inflammation.

We found that some lamellocytes induced by *jumu* deficiency can efficiently engulf latex beads after infection. Lamellocytes are formed in response to wasp infection and are mainly involved in the encapsulation and melanization of foreign pathogens that are too large to undergo phagocytosis [7, 52], although their role in phagocytosis has not been extensively investigated. Previous studies have shown that wasp infection induces a two-lineage model of lamellocyte hematopoiesis: one lineage of lamellocytes is derived from the direct transdifferentiation of plasmatocytes, while the other is a designated lamellocyte lineage, referred to as lamelloblasts [53, 54]. Moreover, the lamellocytes generated from plasmatocytes, expressing the plasmatocyte-specific marker NimC1, exhibit phagocytosis, but the terminally differentiated large lamellocytes without NimC1 expression do not engulf any bacteria [54]. Therefore, the lamellocytes exhibiting phagocytosis among *jumu*-defective mutants may be derived from plasmatocytes. Furthermore, *jumu* knockdown in plasmatocytes (*Hml > GFP > jumu RNAi*) can induce the expression of the lamellocyte marker L1, and the resultant round cells showing coexpression of Hml > GFP and L1 are similar to the activated plasmatocytes induced by wasp infection described in a previous study [53]. These results further suggest that loss of *jumu* can lead to the transdifferentiation of plasmatocytes into lamellocytes in a manner that is similar to immune induction.

### Severe deficiency of *jumu* affects cell mitosis

Our results indicate that Jumu maintains proper hemocyte proliferation and division by regulating the cell cycle, spindle formation and cytokinesis. The loss of *jumu* affects the transcription levels of the *Cyclin* genes *CycA*, *CycB* and *CycD* and the cell cycle- and division-associated genes *png* and *piwi* (Fig.9c)*.* CycA is essential for the control of the cell cycle at the G2/M transition, and the CycA-Cdk1 complex can trigger entry into the S phase [55, 56]. CycB degradation is required for entry into anaphase, and the expression of a stable version of *Drosophila* CycB blocks cytokinesis along with numerous events of mitotic exit [55, 57]. Moreover, CycB is also required for normal spindle formation [58]. The CycD/Cdk4 complex stimulates both cell cycle progression and cell growth, and the overexpression of CycD/Cdk4 leads to larger ommatidia and an enlargement and rough appearance of the eye [59, 60]. According to these results, we speculate that the reduced expression of *CycA* and the increased expression of *CycB* and *CycD* likely simultaneously contribute to the defect in the cell cycle and cytokinesis, consequently causing the generation of enlarged multinucleated hemocytes in *jumu* mutants. PNG is required to repress DNA replication and for proper coupling of the S and M phases in early embryos, and mutation of *png* leads to inappropriate DNA replication and results in large polyploidy nuclei [61, 62]. Thus, the multinucleated phenotype observed in the *jumu*-deficient hemocytes may also be associated with decreased *png* levels. Moreover, the PNG kinase complex regulates the translation of CycB, and the phosphorylation of GNU by CyclinB/CDK1 can also block the activation of the PNG [63, 64]. Moreover, PIWI plays a critical role in the maintenance of cell cycle progression during early embryogenesis; loss of *piwi* leads to severe mitotic defects, including abnormal nuclear morphology, cell cycle arrest and asynchronous nuclear division [65]. The mechanism by which Jumu regulates the expression levels of *CycA*, *CycB*, *CycD*, *png* and *piwi* and the regulatory mechanism of these elements in the control of the cell cycle and division of hemocytes remain to be addressed.

Moreover, previous studies have suggested that activation or overexpression of Dorsal/Dif in hemocytes can promote proliferation [66]. Thus, we speculate that the higher mitotic index observed in the *jumu* double heterozygotes may also be associated with the activation of the Toll signaling pathway. However, we found that the activation of Toll signaling in hemocytes did not result in enlarged hemocytes (data not shown), which suggests that the cell enlargement phenotype caused by the lack of *jumu* is not related to the Toll signaling pathway. The regulatory mechanisms of Jumu in hemocyte proliferation and development remain to be elucidated.

In this study, we also found that mitosis could severely impact phagocytosis (Fig.6f and h), this might be because most microtubule and actin filament participate in the formations of spindle and contractile ring during mitosis, and are not enough to involve phagocytosis. However, most enlarged PH3-positive cells in *jumu*^*GE27806*^*/Df(3R)Exel6157* are mitotic defect, thus, they might have free microtubule and actin filament to participate in cytoskeletal reorganization during phagocytosis (Fig.6g, asterisk). How the cooperation between microtubule and actin filament mediate phagocytosis remain to be addressed.

## Conclusion

Taken together, our findings in this study suggest that Jumu is required for larval circulating hemocyte development as well as phagocytosis and filopodium formation. The severe deficiency of *jumu* induces the generation of lamellocytes through activation of the Toll signal pathway. Furthermore, Jumu regulates hemocyte phagocytosis by affecting the expression of NimC1 and cytoskeletal reorganization.

## Additional files

Additional file 1: **Table S1.** PCR primer sequences. (DOCX 18 kb)

Additional file 2: **Figure S1.** Loss of jumu affects circulating hemocyte differentiation. **Figure S2.** Subcellular localization and expression levels of Ena, Fascin, Rho1 and Profilin in circulating hemocytes. **Figure S3.** Expression levels of Profilin and Rho1 are unchanged in jumu or NimC1 knockdown hemocytes. **Figure S4.** The expression of NimC1 and the subcellular localization of Ena, Fascin, Rho1 or Profilin. **Figure S5.** Loss of jumu does not cause DNA overreplication or cell apoptosis. **Figure S6.** Analysis of the activation of JAK/STAT or JNK signaling pathways. (PDF 14445 kb)

## Abbreviations

AMPs: Antimicrobial peptides
BrdU: 5-Bromo-2-deoxyUridine
JAK/STAT: Janus Kinase/ signal transducer and activator of transcription
JNK: c-Jun N-terminal kinase
MARCM: Mosaic analysis with a repressible cell marker
PBS: Phosphate Buffered Saline
PH3: phospho-histone H3
PI: Phagocytosis index
TUNEL: TdT-mediated dUTP Nick-End Labeling
