# Supplementary material for: Jumu is required for circulating hemocyte differentiation and phagocytosis in Drosophila
Source: Cell Commun Signal. 2018 Dec 5;16:95. doi: 10.1186/s12964-018-0305-3 (PMC6280549; doi:10.1186/s12964-018-0305-3)
Supplement: Supplementary file 1 — Table S1. PCR primer sequences. (DOCX 18 kb) [file 12964_2018_305_MOESM1_ESM.docx]

**Table S1 PCR primer sequences**

| ***target gene*** | ***Forward Primer (5' to 3')*** | ***Reverse Primer (5'to 3')*** |
| --- | --- | --- |
| *jumu* | GATAAGGAGTCAGTGCGTCG | AGTTAATGTCGACGCGAGC |
| *NimC1* | ATACGATGGGATGGGTACTTTC | GTTAGACGATCCAGTTCGGTCAG |
| *eater* | CCCAAGCCAAAGTCCAAATC | GGACAAACGGCCAAGCAG |
| *PGRP-LC* | TCCAAACACGAAGATCCGG | TGGTTCAACGTCTTTCCGAAG |
| *crq* | GAGACCAGCGCAAGATCGTC | TGAATAGTAAACAAGCCATCGTAAG |
| *Sr-CI* | CTGGAAGCGAGTCAGCACG | CATGAAGTAGTGGAATCGAAAGC |
| *Dscam* | TGATGTAAAGGCTCTTGTTATGGG | CTCGTAGGGCTTGTTCTTCTCC |
| *peste* | AATATCGGACCCTGCTCCTTT | CGTCGGGACATTCTCATACAAG |
| *draper* | GCGTGCATAATCTTCGCAGT | CTCATCGTGCTCCTCTGGTC |
| *ena* | GATCCCGATCCTGAAGCC | CGCCACTGTTCGTTGTGTT |
| *fascin* | AACCAGGACGAGGTCGGT | CGAGGTGGCAAACAGATGAC |
| *profilin* | AGCAGGACGGTCTCACCAG | CCGCAAGTAATCAGATAATCTC |
| *rho1* | CAGTGGATTCACCCGATTC | GCATCTGGTCTTCTTCCTCTTT |
| *CycA* | TTGATTGGCTGGTTGAGGTTTC | GATTTCCTCGTATTTTGCGGC |
| *CycB* | ACGCCCACTCTGACCTTCT | TGGCTCTGGCCCACAATC |
| *CycD* | AAATTGCCAGGAAGAGGTC | GAAGTCCAAGGGAGTCACAGAT |
| *CycE* | ATACATTTAGTCGGGAGATGGCTT | GTTTGGATAATGTGCGAGTCGT |
| *pav* | CGCAAATCCGTAACGAAACT | GCTTGAGGCACTCCACATC |
| *png* | CTGATGGGCTACTTCCGC | CGACTTGAAATCCACCTTGC |
| *bam* | GTGAAGAGCACGATGCCTAAG | CAAATAGGATGCGAAACCCT |
| *piwi* | AACGACCAAGAACCGTAGC | GCCAAAGGGTGTTAGCGAG |
| *hop* | GAGCAGGTTGCCATCAAGA | CGTGGATTATTGAGATTGGGT |
| *Stat92E* | GAAACTCCAGCGTGAGGAG | CATAGACTGAACGCAGCAGAT |
| *puc* | CATGTGGCTAGCAATTTGA | GTCTTCGAAAAACGTACAGC |
| *Bsk* | GACAGCTCAGCACCAACACT | GCTTGGCATGGGTTACATTT |
| *P1A2* | TGGAGTGGTTGGCAGACAAA |  |
| *P1isoBfw* | GTCATTGTCCTTTGGCACAA |  |
| *P1del.utanA,B* |  | ACGTTGCACTCGCACACATT |
| *RpL32* | AGTCGGATCGATATGCTAAGCTGT | TAACCGATGTTGGGCATCAGATACT |
