# Supplementary material for: Jumu is required for circulating hemocyte differentiation and phagocytosis in Drosophila
Source: Cell Commun Signal. 2018 Dec 5;16:95. doi: 10.1186/s12964-018-0305-3 (PMC6280549; doi:10.1186/s12964-018-0305-3)
Supplement: Supplementary file 2 — Figure S1. Loss of jumu affects circulating hemocyte differentiation. Figure S2. Subcellular localization and expression levels of Ena, Fascin, Rho1 and Profilin in circulating hemocytes. Figure S3. Expression levels of Profilin and Rho1 are unchanged in jumu or NimC1 knockdown hemocytes. Figure S4. The expression of NimC1 and the subcellular localization of Ena, Fascin, Rho1 or Profilin. Figure S5. Loss of jumu does not cause DNA overreplication or cell apoptosis. Figure S6. Analysis of the activation of JAK/STAT or JNK signaling pathways. (PDF 14445 kb) [file 12964_2018_305_MOESM2_ESM.pdf]

Supplemental Figures:

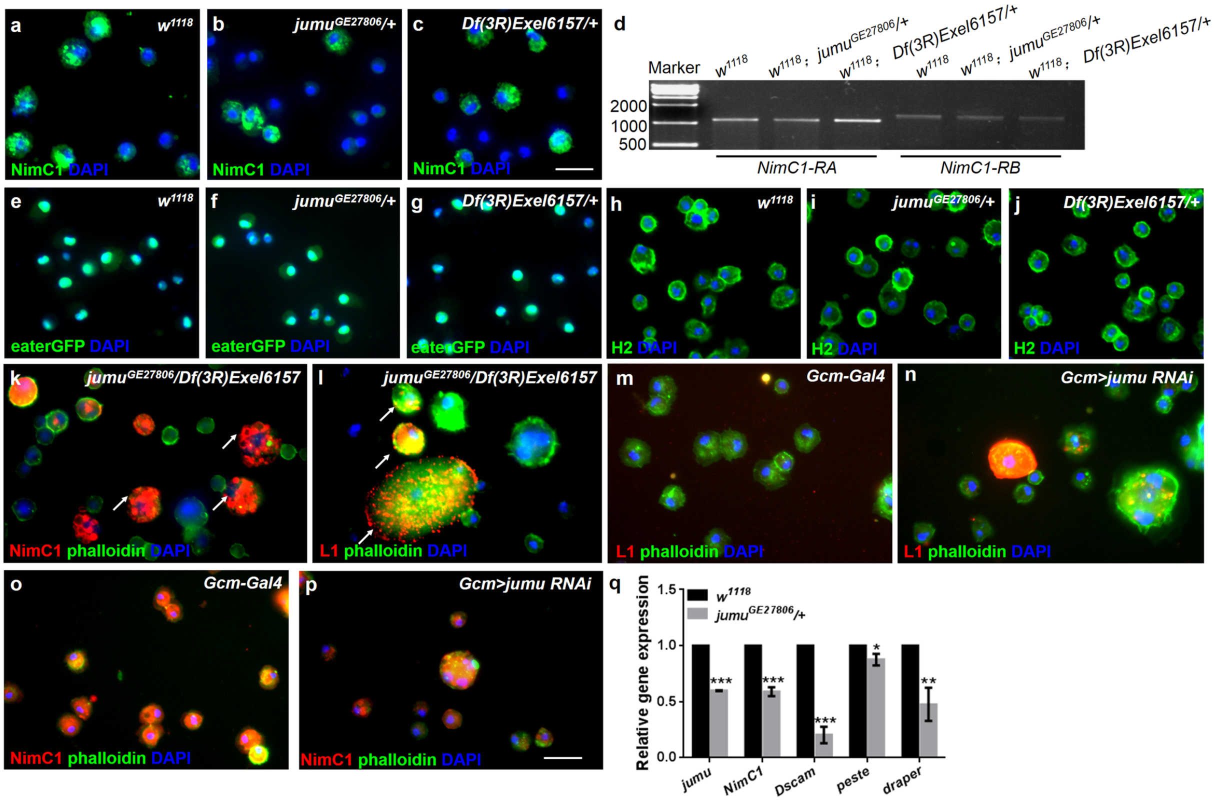

Figure S1. Loss of *jumu* affects circulating hemocyte differentiation. a-c Immunostaining against NimC1 (green) shows that part of the circulating hemocytes of *jumu* heterozygotes do not express NimC1. **d** Agarose gel electrophoresis of RT-PCR products obtained using primer pairs specific for *NimC1-RA* (1138 bp) and *NimC1-RB* (1268 bp). We detected the appropriately sized fragment in all investigated lines. **e-j** The expression levels of eaterGFP and H2 in *jumu* heterozygote circulating hemocytes are similar to those in controls. **k** Immunostaining against NimC1 (red) and phalloidin staining (green) shows that only 50% of the circulating hemocytes of *jumu* double heterozygotes express NimC1, and nearly 60% of the enlarged cells are labeled with an anti-NimC1 antibody (arrows). **l** Immunostaining against L1 (red) and phalloidin staining (green) shows that more than 10% of the lamellocytes are observable in *jumu*

double heterozygotes circulating hemocytes (arrows). **m, n** Immunostaining against L1 (red) shows that nearly 10% of the lamellocytes are observable in *Gcm>jumu RNAi* circulating hemocytes. **o, p** Immunostaining against NimC1 (red) shows that NimC1 expression is reduced in *Gcm>jumu RNAi* circulating hemocytes compared with that in controls. **q** Real-time PCR analysis of phagocytosis receptor gene levels in circulating hemocytes. Error bars represent the S.E.M of at least 3 independent experiments; *P<0.05; **P<0.01; ***P<0.001 (Student's *t*-test). Scale bars: 20 μm.

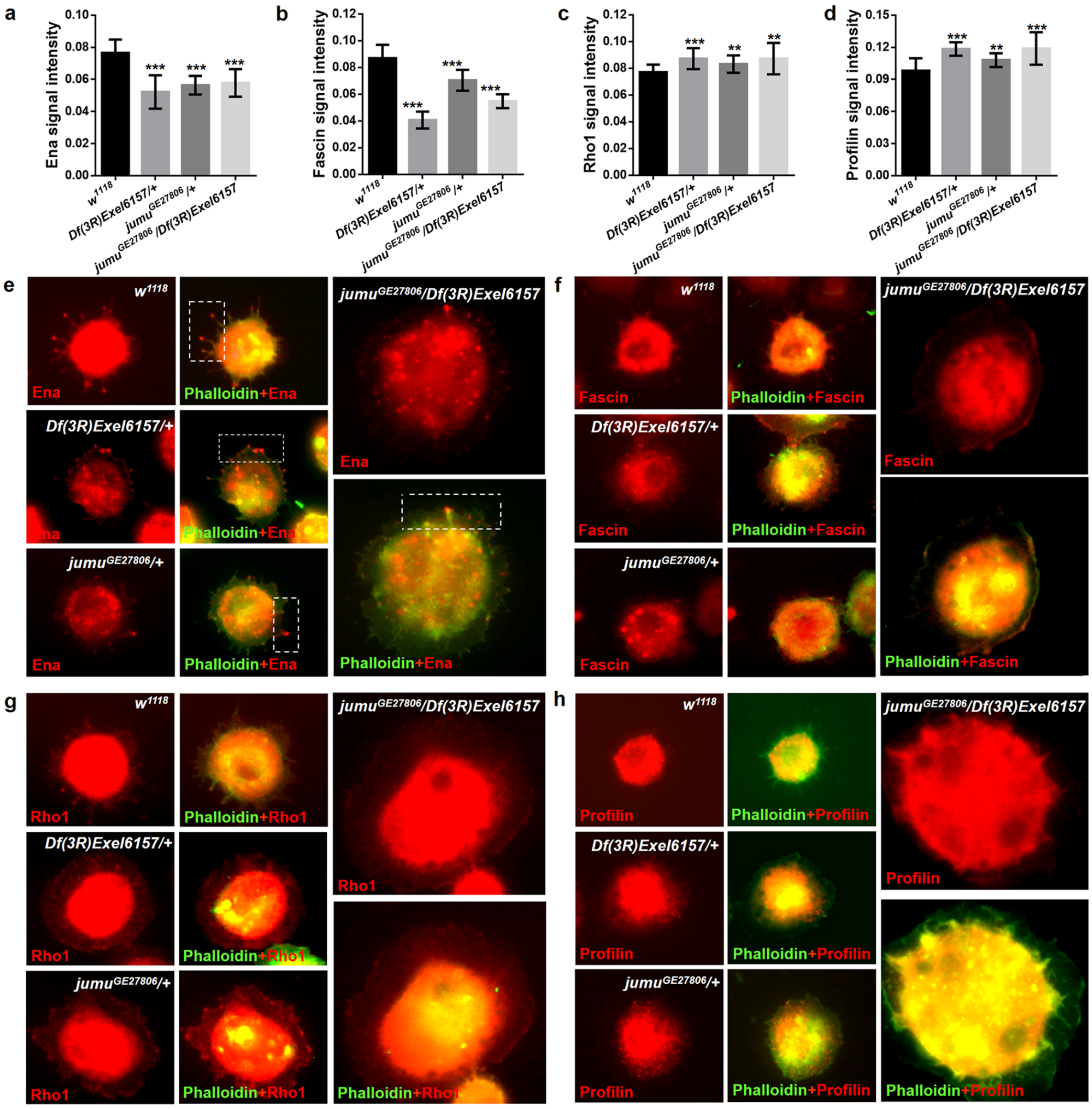

Figure S2. Subcellular localization and expression levels of Ena, Fascin, Rho1 and Profilin in circulating hemocytes. a-d Quantification of signal intensities of Ena, Fascin, Rho1 or Profilin. **e-h** Immunostaining against Ena (red), Fascin (red), Rho1 (red) or Profilin (red) and phalloidin staining (green) shows the subcellular localization of Ena, Fascin, Rho1 and Profilin in control and *jumu* mutants circulating hemocytes. Error bars represent the S.E.M of at least 3 independent experiments; $^{**}P<0.01$; $^{***}P<0.001$ (Student's *t*-test). Scale bars: 10 μm.

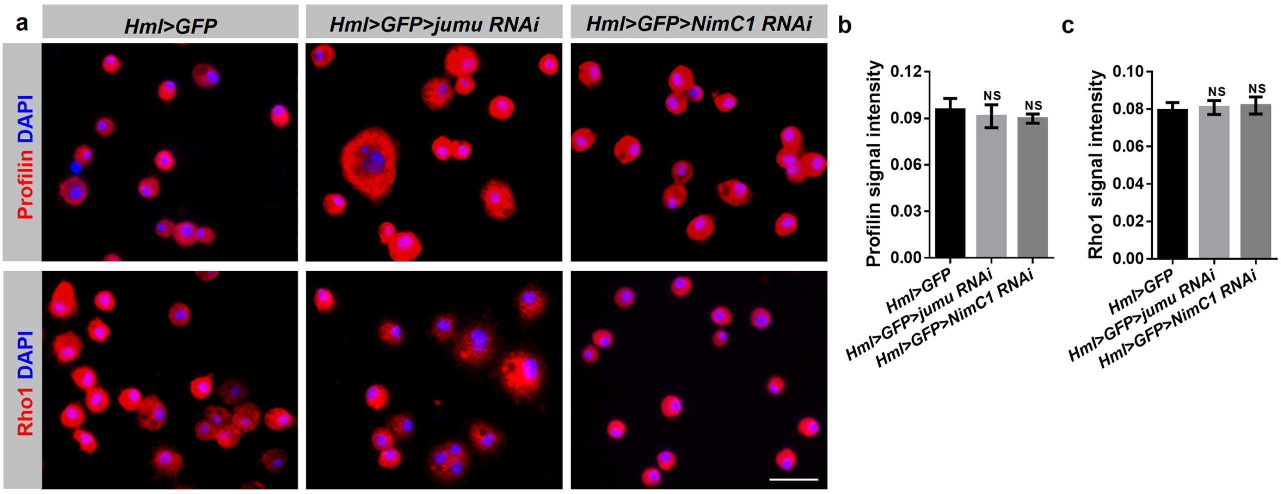

Figure S3. Expression levels of Profilin and Rho1 are unchanged in *jumu* or *NimC1* knockdown hemocytes. a Immunostaining against Profilin (red) and Rho1 (red) in *jumu* or *NimC1* knockdown hemocytes. **b, c** Quantification of signal intensities of Profilin and Rho1. Error bars represent the S.E.M of at least 3 independent experiments; NS, not significant (Student's *t*-test). Scale bars: 20 μm.

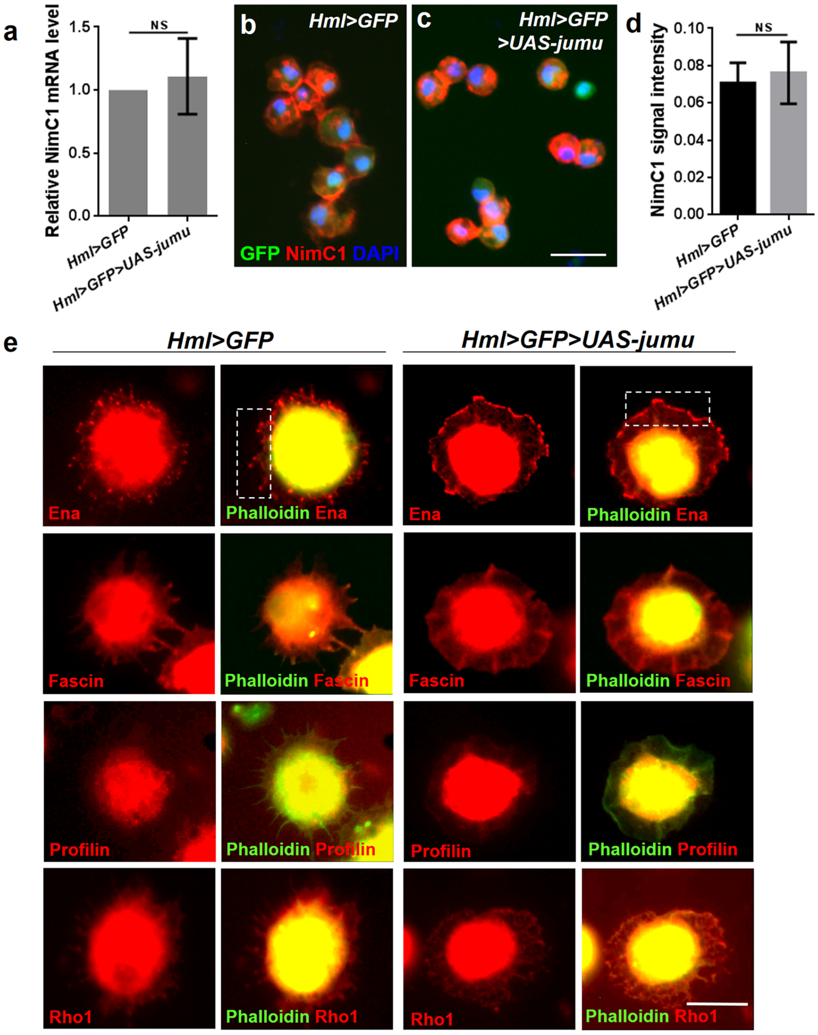

Figure S4. The expression of NimC1 and the subcellular localization of Ena, Fascin, Rho1 or Profilin. a Real-time PCR analysis of *NimC1* levels in circulating hemocytes. **b, c** Immunostaining against NimC1 (red) shows that NimC1 expression is unchanged in *Hml>GFP>UAS-jumu* circulating hemocytes compared with that in the controls. **d** Quantification of the signal intensities of NimC1. **e** Immunostaining against Ena, Fascin, Rho1 or Profilin and phalloidin staining (green) show the subcellular localization of Ena, Fascin, Rho1 and Profilin in control and *Hml>GFP>UAS-jumu* circulating hemocytes. Error bars represent the S.E.M of at least 3 independent experiments; NS, not significant (Student's *t*-test). Scale bars: 20 μm (**b, c**); 10 μm (**e**).

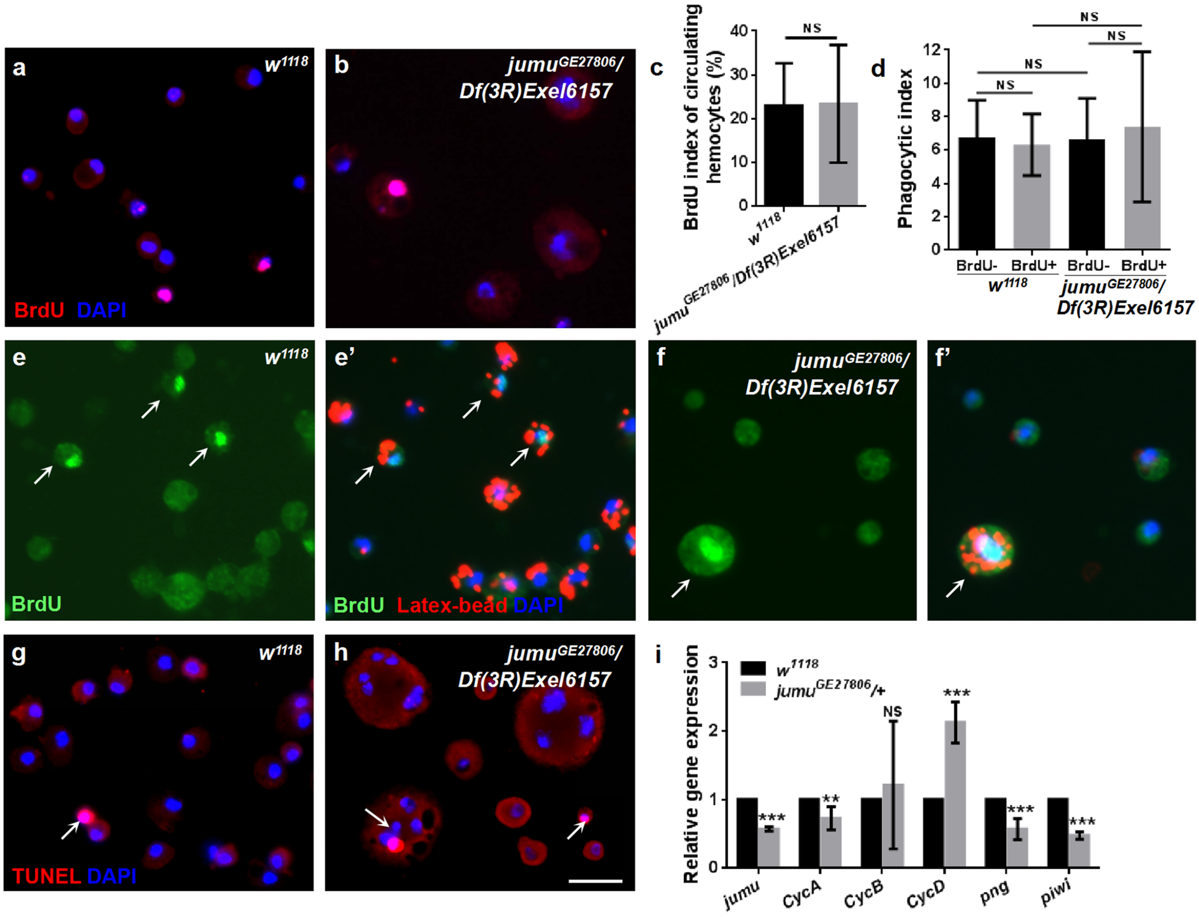

Figure S5. Loss of *jumu* does not cause DNA overreplication or cell apoptosis. a, b Cells in the S phase from the third-instar larval circulating hemocytes are analyzed using BrdU incorporation (red); the amount of BrdU incorporation is not increased in the *jumu* double heterozygotes compared with that in the controls. **c** Quantification of the BrdU index. **d** Quantification of phagocytic indexes of latex beads. **e-f'** Immunostaining against BrdU (green) in circulating hemocytes isolated from third-instar larvae injected with latex beads (red). **g, h** Apoptosis is analyzed through TUNEL assays in circulating cells. **i** Real-time PCR analysis of genes associated with the cell cycle and division levels of *jumu* mutant hemocytes. Error bars represent the S.E.M of at least 3 independent experiments; NS, not significant; $^{**}P<0.01$; $^{***}P<0.001$ (Student's *t*-test in **c**, I; one-way ANOVA in **d**). Scale bars: 20 µm.

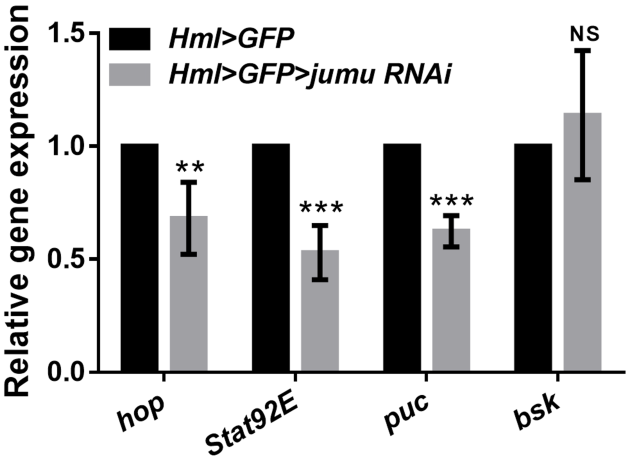

Figure S6. Analysis of the activation of JAK/STAT or JNK signaling pathways.

Real-time PCR analysis of target genes of JAK/STAT or JNK levels from *jumu* knockdown hemocytes. Error bars represent ±S.E.M for at least 3 independent experiments; NS, not significant; **P<0.01; ***P<0.001 (Student's *t*-test).
